# Genome-Wide Association Study to Identify Common Variants Associated with Brachial Circumference: A Meta-Analysis of 14 Cohorts

**DOI:** 10.1371/journal.pone.0031369

**Published:** 2012-03-29

**Authors:** Vesna Boraska, Aaron Day-Williams, Christopher S. Franklin, Katherine S. Elliott, Kalliope Panoutsopoulou, Ioanna Tachmazidou, Eva Albrecht, Stefania Bandinelli, Lawrence J. Beilin, Murielle Bochud, Gemma Cadby, Florian Ernst, David M. Evans, Caroline Hayward, Andrew A. Hicks, Jennifer Huffman, Cornelia Huth, Alan L. James, Norman Klopp, Ivana Kolcic, Zoltán Kutalik, Debbie A. Lawlor, Arthur W. Musk, Marina Pehlic, Craig E. Pennell, John R. B. Perry, Annette Peters, Ozren Polasek, Beate St Pourcain, Susan M. Ring, Erika Salvi, Sabine Schipf, Jan A. Staessen, Alexander Teumer, Nicholas Timpson, Veronique Vitart, Nicole M. Warrington, Hanieh Yaghootkar, Tatijana Zemunik, Lina Zgaga, Ping An, Verneri Anttila, Ingrid B. Borecki, Jostein Holmen, Ioanna Ntalla, Aarno Palotie, Kirsi H. Pietiläinen, Juho Wedenoja, Bendik S. Winsvold, George V. Dedoussis, Jaakko Kaprio, Michael A. Province, John-Anker Zwart, Michel Burnier, Harry Campbell, Daniele Cusi, George Davey Smith, Timothy M. Frayling, Christian Gieger, Lyle J. Palmer, Peter P. Pramstaller, Igor Rudan, Henry Völzke, H. -Erich Wichmann, Alan F. Wright, Eleftheria Zeggini

**Affiliations:** 1 Wellcome Trust Sanger Institute, The Morgan Building, Wellcome Trust Genome Campus, Hinxton, Cambridge, United Kingdom; 2 Department of Medical Biology, University of Split School of Medicine, Split, Croatia; 3 Wellcome Trust Centre for Human Genetics, University of Oxford, Oxford, United Kingdom; 4 Institute of Genetic Epidemiology, Helmholtz Zentrum München - German Research Center for Environmental Health, Neuherberg, Germany; 5 Geriatric Unit, Azienda Sanitaria di Firenze, Florence, Italy; 6 School of Medicine and Pharmacology, The University of Western Australia, Perth, Australia; 7 Institute of Social and Preventive Medicine, Centre Hospitalier Universitaire Vaudois and University of Lausanne, Lausanne, Switzerland; 8 Genetic Epidemiology and Biostatistics Platform, Ontario Institute for Cancer Research, Toronto, Canada; 9 Interfaculty Institute for Genetics and Functional Genomics, University of Greifswald, Greifswald, Germany; 10 MRC CAiTE Centre, School of Social and Community Medicine, University of Bristol, Bristol, United Kingdom; 11 MRC Human Genetics Unit, Institute of Genetics and Molecular Medicine, Edinburgh, United Kingdom; 12 Center for Biomedicine, European Academy Bozen/Bolzano (EURAC), Bolzano, Italy, Affiliated Institute of the University of Lübeck, Lübeck, Germany; 13 Institute of Epidemiology II, Helmholtz Zentrum München - German Research Center for Environmental Health, Neuherberg, Germany; 14 Busselton Population Medical Research Foundation, Sir Charles Gairdner Hospital, Nedlands, Western Australia, Australia; 15 Department of Pulmonary Physiology/West Australian Sleep Disorders Research Institute, Sir Charles Gairdner Hospital, Nedlands, Western Australia, Australia; 16 Unit for Molecular Epidemiology, Helmholtz Zentrum München - German Research Center for Environmental Health, Neuherberg, Germany; 17 Croatian Centre for Global Health, University of Split School of Medicine, Split, Croatia; 18 Department of Medical Genetics, University of Lausanne, Lausanne, Switzerland; 19 Department of Respiratory Medicine, Sir Charles Gairdner Hospital, Nedlands, Western Australia, Australia; 20 Schools of Population Health and Medicine and Pharmacology, University of Western Australia, Perth, Western Australia, Australia; 21 School of Women's and Infants' Health, The University of Western Australia, Perth, Western Australia, Australia; 22 Genetics of Complex Traits, Peninsula Medical School, University of Exeter, Exeter, United Kingdom; 23 School of Social and Community Medicine, University of Bristol, Bristol, United Kingdom; 24 Department of Medicine, Surgery and Dentistry, University of Milano, Milano, Italy; 25 Genomics and Bioinformatics Platform, Fondazione Filarete, University of Milano, Milano, Italy; 26 Institute for Community Medicine/SHIP-Clinical Epidemiological Research, University of Greifswald, Greifswald, Germany; 27 Studies Coordinating Centre, Division of Hypertension and Cardiovascular Rehabilitation, Department of Cardiovascular Diseases, University of Leuven, Leuven, Belgium; 28 Department of Epidemiology, Maastricht University, Maastricht, The Netherlands; 29 Centre for Population Health Sciences and Institute of Genetics and Molecular Medicine, College of Medicine and Veterinary Medicine, University of Edinburgh, Edinburgh, United Kingdom; 30 Andrija Štampar School of Public Health, School of Medicine, University of Zagreb, Zagreb, Croatia; 31 Division of Statistical Genomics and Department of Genetics Washington University School of Medicine, St. Louis, Missouri, United States of America; 32 Institute for Molecular Medicine Finland (FIMM), University of Helsinki, Helsinki, Finland; 33 HUNT Research Centre, Department of Public Health and General Practice, Norwegian University of Science and Technology, Levanger, Norway; 34 Harokopio University of Athens, Department of Dietetics and Nutrition, Athens, Greece; 35 Department of Medical Genetics, University and University Central Hospital of Helsinki, Helsinki, Finland; 36 The Broad Institute of MIT and Harvard, Cambridge, Massachusetts, United States of America; 37 Obesity Research Unit, Department of Medicine, Division of Internal Medicine, Helsinki University Central Hospital, Helsinki, Finland; 38 Department of Public Health, Hjelt Institute, University of Helsinki, Helsinki, Finland; 39 Department of Neurology, Oslo University Hospital and University of Oslo, Oslo, Norway; 40 National Institute for Health and Welfare, Dept of Mental Health and Substance Abuse Services, Helsinki, Finland; 41 Service of Nephrology, University Hospital of Lausanne (CHUV), Lausanne, Switzerland; 42 Division of Nephrology, San Paolo Hospital, Milano, Italy; 43 Prosserman Centre for Health Research, Samuel Lunenfeld Research Institute, Toronto, Canada; 44 Department of Neurology, General Central Hospital, Bolzano, Italy; 45 Department of Neurology, University of Lübeck, Lübeck, Germany; 46 Institute of Epidemiology I, Helmholtz Zentrum München - German Research Center for Environmental Health, Neuherberg, Germany; 47 Institute of Medical Informatics, Biometry and Epidemiology, Chair of Epidemiology, Ludwig-Maximilians-Universität, Munich, Germany; 48 Klinikum Grosshadern, Munich, Germany; University of Hong Kong, Hong Kong

## Abstract

Brachial circumference (BC), also known as upper arm or mid arm circumference, can be used as an indicator of muscle mass and fat tissue, which are distributed differently in men and women. Analysis of anthropometric measures of peripheral fat distribution such as BC could help in understanding the complex pathophysiology behind overweight and obesity. The purpose of this study is to identify genetic variants associated with BC through a large-scale genome-wide association scan (GWAS) meta-analysis. We used fixed-effects meta-analysis to synthesise summary results across 14 GWAS discovery and 4 replication cohorts comprising overall 22,376 individuals (12,031 women and 10,345 men) of European ancestry. Individual analyses were carried out for men, women, and combined across sexes using linear regression and an additive genetic model: adjusted for age and adjusted for age and BMI. We prioritised signals for follow-up in two-stages. We did not detect any signals reaching genome-wide significance. The *FTO* rs9939609 SNP showed nominal evidence for association (p<0.05) in the age-adjusted strata for men and across both sexes. In this first GWAS meta-analysis for BC to date, we have not identified any genome-wide significant signals and do not observe robust association of previously established obesity loci with BC. Large-scale collaborations will be necessary to achieve higher power to detect loci underlying BC.

## Introduction

Brachial circumference (BC) is a composite measure of muscle mass, skeletal size and fat tissue [1], [2]. BC has been widely used in epidemiological and clinical studies as a proxy for body composition [3]. Analysis of anthropometric measures of peripheral fat distribution like BC could help in understanding complex phenotypes such as overweight and obesity that can lead to the development of chronic diseases, for example type 2 diabetes (T2D) and cardiovascular disease [4], [5], [6]. Research of upper and lower body fat association with diabetes in families of African origin suggested that arm and leg fat could be used as obesity-related phenotypes in association studies [7]. Obesity in children can also lead to development of chronic diseases such as hyperlipidaemia, hyperinsulinemia, hypertension, and early atherosclerosis later on in life [8]. It was shown that BC closely reflects body fat mass in children and adolescents and its use was recommended as a screening method for prediction of obesity and overweight [3], [8]. Moreover, BC has been used for decades for the assessment of nutritional status of children in developing countries and has also been proposed as a tool for monitoring nutritional status and weight in the elderly [3], [9], [10], [11]. Peripheral and overall fat distribution, assessed through body mass index (BMI), is partly modulated through different genetic effects [12].

There are differences in the amount and distribution pattern of soft tissue between sexes. In general, men have higher total body lean tissue and lower percent body fat whereas women have higher total body fat and a lower proportion of lean tissue in the upper body [1], [13], [14], [15], [16]. Women have more subcutaneous fat than men over the buttocks and thighs and behind the upper arms [17]. In addition, it was recently shown that diabetic women of African ancestry have a higher proportion of fat deposited in their arms than diabetic men [7]. Due to the effects of sex hormones but also due to heavier physical activity and involvement in more power sports, men have larger muscle size/mass and larger BC compared to women [17], [18]. This may indicate that BC is a better measure of muscularity in men and adipose tissue in women. Analysis of the genetic contribution to BC in Belgian nuclear families indicated that BC is influenced by additive genetic effects (h^2^ = 0.57) [19]. Miljkovic-Gacic et al also estimated high heritability of upper arm body fat storage and also pointed that genetic factors play a role in defining sexual dimorphism of lower body fat distribution in individuals of African origin [7]. Genetic effects on fat distribution can be related to sex and in this study we aimed to evaluate sex-specific genetic associations with BC through analysis of men and women separately, as well as common associations through the analysis of a combined dataset.

Weight gain and redistribution of fat tissue, the main characteristics of aging, influence body composition and consequently affect BC [20]. The decrease in BC that is observed in elderly men and women points to substantial subcutaneous fat loss and redistribution of fat from extremity to trunk [4]. Additionally, aging is also characterised by loss in skeletal muscle mass, known as sarcopenia [21], [22]. To account for these effects of aging on BC we adjusted all our analyses for the age of individuals.

In summary, BC is a measure of both adiposity and muscularity [1]. This study aimed to identify shared and sex-specific genetic variants associated with BC through a large-scale genome-wide association scan (GWAS) meta-analysis.

## Materials and Methods

### Discovery dataset: sample characteristics

We conducted genome-wide meta-analysis across 14 discovery datasets, comprising a total of 18,753 individuals (8,961 men, 9,792 women) of European ancestry. BC measurement was taken uniformly across studies; it was measured in mm using non-elastic tape that was wrapped around the upper arm, at the medium of the upper arm length i.e. at the midpoint between the acromion and the olecranon. BC was normally distributed. Individuals that had BC higher or lower than 3 standard deviations from the mean were removed from each dataset prior to conducting association analysis. Sample characteristics across all datasets are presented in Table 1. Detailed sample characteristics on men and women separately are presented in Table S1. A bar chart of BC measures across studies is presented in Figure S1.

**Table 1:** Contributing studies and sample characteristics.

Cohort	Sample (% female)	Age, years Mean (stdev)	BC, mm Mean (stdev)	BMI, kg/m^2^ Mean (stdev)	Correlation BC and BMI	Ancestry	PMID number/Reference
DISCOVERY DATASET							
ALSPAC	4428 (51.2)	13.77 (0.21)	250.03 (31.93)	20.22 (3.25)	0.916	UK	11237119
SHIP	4070 (50.8)	49.73 (16.27)	291.61 (33.17)	27.31 (4.77)	0.759	Germany	20167617
KORA S4	1788 (51.3)	53.78 (8.89)	298.79 (29.87)	27.60 (4.30)	0.764	Germany	16032514
KORA S3	1634 (50.4)	52.65 (10.08)	288.96 (27.33)	27.28 (4.03)	0.762	Germany	16032514
InCHIANTI	1169 (55.3)	68.09 (15.38)	289.54 (32.67)	27.19 (4.15)	0.681	Italy	11129752, 18464913
BUSSELTON	924 (57.4)	54.15 (17.19)	314.51 (36.62)	25.97 (4.08)	0.842	Australia	/
CROATIA-VIS	905 (57.6)	56.27 (15.52)	311.12 (34.22)	27.28 (4.18)	0.775	Croatia	8327257
MICROS	895 (45.1)	44.99 (16.82)	285.70 (32.84)	25.34 (4.64)	0.721	Italy	17550581
RAINE	884 (48.6)	17.03 (0.23)	274.2 (34.91)	23.07 (4.37)	0.841	Australia	8105165, 9224128, 8855394
CROATIA-KORCULA	841 (63.8)	56.28 (13.98)	333.72 (47.20)	27.91 (4.19)	0.592	Croatia	19260141
CROATIA-SPLIT	495 (57.6)	49.04 (14.65)	310.30 (36.26)	26.93 (4.19)	0.857	Croatia	19260138
CoLaus-Hercules	369 (47.7)	56.93 (10.31)	283.19 (29.41)	25.89 (4.15)	0.82	Switzerland	17701901, 19543373, 18366642
HYPERGENES-controls	196 (54.6)	59.16 (7.62)	287.4 (27.0)	25.93 (3.42)	0.78	Italy	20935630
HYPERGENES-cases	155 (52.9)	41.02 (8.77)	300.8 (35.3)	27.38 (5.16)	0.77	Italy	20935630
REPLICATION STAGE 1							
FamHS (*in silico*)	967 (51.8)	62.5 (11.30)	333.5 (43.5)	29.00 (5.30)	0.806	European Americans	8651220
REPLICATION STAGE 2							
HUNT (*in silico*)	1626 (73.4)	40.9 (12.19)	292.2 (30.12)	26.00 (4.09)	0.81	Norway	Holmen et al. 2003
TEENAGE (*de novo*)	819 (55.4)	13.42 (0.85)	259.03 (33.27)	21.19 (3.45)	0.899	Greece	/
FatTwin (*in silico*)	211 (42.7)	27.65 (2.13)	317.48 (41.11)	25.15 (4.54)	0.856	Finland	17254406, 19584879

### Ethics Statement

Each study obtained ethical approval from their respective research ethics committee and all participants gave signed informed consent in accordance with the Declaration of Helsinki.

### Genotyping, imputation and quality control

All samples were genotyped using commercially available Illumina (Illumina, Inc., San Diego, CA, USA) or Affymetrix (Affymetrix, Inc., Santa Clara, CA, USA) platforms. Imputation of untyped variants was based on HapMap Phase II data for the CEU population. Quality control (QC) of directly typed and imputed variants was conducted separately in each study. Study-specific information on genotyping platform, imputation method and QC metrics is presented in Table S2.

### Genome-wide association analysis in contributing studies

Association analysis was performed in each study separately. Analyses were performed for directly typed and imputed autosomal variants using linear regression and an additive genetic model, adjusted for age and adjusted for age and BMI, in men and women separately. Specifically, four types of analyses were carried out: women – age adjusted, women – age and BMI adjusted, men - age adjusted, men – age and BMI adjusted. By adjusting a set of our analyses with BMI we aim to differentiate the effects of loci solely influencing regional adiposity and/or muscularity. BC changes throughout the life and by adjusting analyses for age we aim to exclude the effect of age on BC. Association analyses of imputed variants took genotype uncertainty into account. Where necessary, the first three genotype-based principal components were used as covariates. Studies with related individuals additionally adjusted analyses for family relatedness using linear mixed models. Association analysis software for each study is presented in Table S2. Sensitivity analysis excluding the two studies comprised of adolescent individuals (ALSPAC and RAINE) was also carried out, to exclude potential effects of genes that might be involved in body development and growth during adolescence. We also investigated the effects of 54 previously established genetic loci associated with overall adiposity, assessed through BMI and other measures of fat distribution such as waist-to-hip ratio, and peripheral adiposity, and of 64 previously established T2D loci and 41 loci influencing glycaemic traits with BC in discovery dataset.

### GWAS meta-analysis

We performed six fixed-effects meta-analyses to synthesize summary statistics across 14 datasets using GWAMA [23]. Meta-analyses were performed separately in men and women but also in the combined set. Prior to meta-analysis we excluded SNPs with minor allele frequency (MAF) lower than 0.05 and SNPs with low imputation accuracy scores. We used a cut-off of rsq_hat <0.3 for genotypes imputed with MACH [24] software and a cut-off of proper info score <0.5 for IMPUTE [25] software. The number of directly genotyped and imputed SNPs that passed QC criteria across all studies and were subsequently meta-analysed is presented for each meta-analysis in Table S3. The genomic control (GC) inflation factor was calculated and applied to the results for each study separately, prior to the meta-analysis. The meta-analysis results were also corrected for overall GC. We investigated evidence of heterogeneity using the I^2^ statistic [26]. We created quantile-quantile (QQ) and Manhattan plots to visualise genome-wide association results.

### Replication stage 1

**Figure 1:**
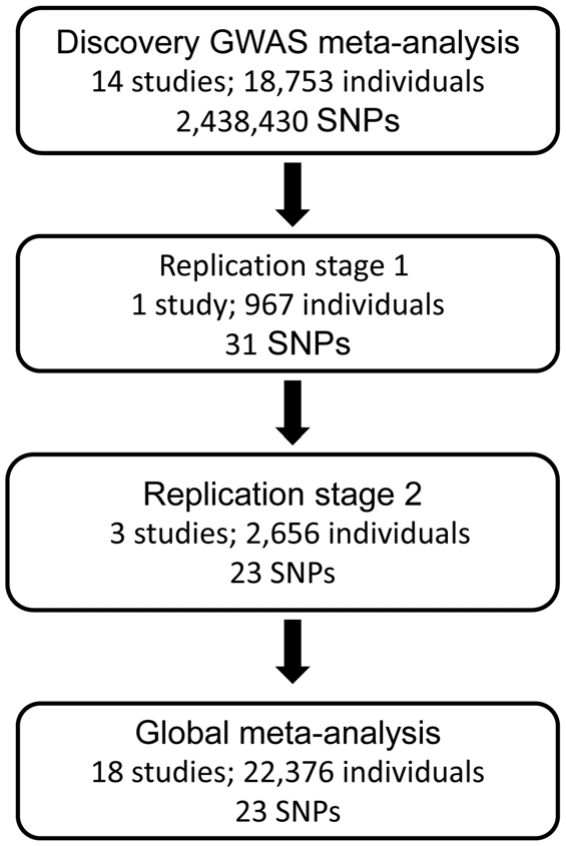
Study design. Each step includes non-overlapping, independent datasets.

On the basis of the GWAS meta-analysis results we conducted a two-stage follow-up of prioritised SNPs in independent datasets. The study design is presented in Figure 1.

We selected 31 SNPs for initial *in silico* replication: we focused on SNPs with p-values below or close to 10^−5^ and additionally examined their genomic location, prioritising those within or near biologically interesting genes. When there were many SNPs with p-values below 10^−5^ in the same genomic region we selected the two most significant variants for follow-up. We visually inspected cluster plots (in studies with available intensity data) for all selected SNPs prior to replication.

Replication stage 1 samples included 967 *s*ubjects from the Family Heart Study (FamHS) (Table 1 and Table S2) [27]. We combined summary statistics for 31 directly typed and imputed SNPs across the discovery and stage 1 replication studies using fixed-effects meta-analysis. We performed the same sets of analyses as described for the discovery dataset.

### Replication stage 2

On the basis of meta-analysis results across the discovery and replication stage 1 datasets, and taking into account the results of our sensitivity analyses, we prioritised a subset of 22 SNPs for the second round of replication. SNPs were selected primarily on the basis of their overall statistical significance. We also selected the established obesity variant rs9939609 in *FTO* to assess its association with BC in a larger sample size. Overall, 23 SNPs were taken forward for *de novo* genotyping in the Greek TEENs of Attica: Genes & Environment (TEENAGE) study and *in silico* replication in two studies, the Nord-Trøndelag Health Study (HUNT) [28] and TwinFat [29], [30]. The main reason for conducting two stage replication was to refine significance of initially prioritised SNPs using *in silico* data (replication stage 1) and to select a subset of SNPs with greater evidence of association to fit one genotyping multiplex (iPLEX™ Gold Assay) for *de novo* genotyping in the TEENAGE cohort (replication stage 2). Two studies with *in silico* data (HUNT and TwinFat) joined the analysis at the advanced stage of replication stage 2.

*TEENAGE cohort*. The TEENAGE cohort consists of 819 adolescent students attending all three classes of public secondary schools in the Attica region of Greece (Table 1). Genotyping of the 23 prioritised SNPs was performed using the iPLEX™ Gold Assay (Sequenom® Inc.). Assays for two SNPs (rs7837164 and rs4833582) could not be designed, therefore their proxies (rs4831616 and rs10018120 with r^2^ = 1 and r^2^ = 1, respectively) were genotyped instead. Assays for all SNPs were designed using the eXTEND suite and MassARRAY Assay Design software version 3.1 (Sequenom® Inc.). Amplification was performed in a total volume of 5 µL containing ∼0.06–0.4 ng genomic DNA, 100 nM of each PCR primer, 500 µM of each dNTP, 1.25× PCR buffer (Qiagen), 1.625 mM MgCl_2_ and 1 U HotStar Taq® (Qiagen). Reactions were heated to 94°C for 15 min followed by 45 cycles at 94°C for 20 s, 56°C for 30 s and 72°C for 1 min, then a final extension at 72°C for 3 min. Unincorporated dNTPs were SAP digested prior to iPLEX™ Gold allele specific extension with mass-modified ddNTPs using an iPLEX Gold reagent kit (Sequenom® Inc.). SAP digestion and extension were performed according to the manufacturer's instructions with reaction extension primer concentrations adjusted to between 0.7–1.8 µM, dependent upon primer mass. Extension products were desalted and dispensed onto a SpectroCHIP using a MassARRAY Nanodispenser prior to MALDI-TOF analysis with a MassARRAY Analyzer Compact mass spectrometer. Genotypes were automatically assigned and manually confirmed using MassARRAY TyperAnalyzer software version 4.0 (Sequenom® Inc.).

We applied the following sample/SNP QC exclusions for these *de novo* genotype data: sex inconsistencies, sample call rate <98% and exact HWE p-value<0.0001. The average assay call rate was 0.993. Overall, 806 samples and 23 SNPs passed our QC criteria in the TEENAGE replication cohort. Linear regression analysis under an additive genetic model, taking into account age and age and BMI in each stratum (men, women and combined set), was carried out using Plink [31].

*HUNT and TwinFat studies*: The HUNT study consisted of 1626 [28] and TwinFat consisted of 216 individuals [29], [30] (Table 1 and Table S2).

### Global meta-analysis

We performed meta-analysis for the 23 prioritised SNPs across the discovery and all replication datasets, comprising a total of 22,376 individuals (12,031 women and 10,345 men). We performed the same six sets of analyses as described for the discovery dataset. Power for the global meta-analysis was calculated using Quanto [32].

## Results

**Figure 2:**
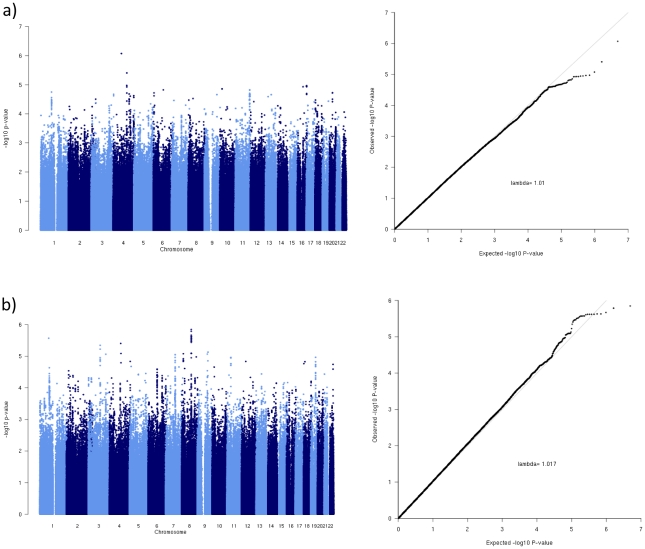
Manhattan and QQ plots based on meta-analyses results of the discovery panel: a) combined set - age adjusted, b) combined set - age and BMI adjusted.

We did not observe an excess of signals in the six meta-analyses that were carried out. QQ and Manhattan plots for combined analysis (adjusted for age and adjusted for age and BMI) are shown in Figure 2. QQ and Manhattan plots for men and women are shown in Figure S2.

Of the 31 SNPs initially taken forward, 4 showed association with p<0.05 in replication set 1. Twenty one of 31 signals had effects in the same direction as the discovery GWAS meta-analysis (binomial p-value = 0.07, given we expect 50% of signals to be in the same direction by chance) (Table S4). Of the 23 SNPs prioritised for stage 2 replication, 12 had effects in the same direction as the GWAS meta-analysis (binomial p-value = 1) and 1 SNP achieved p<0.05 in replication set 2 (Table S4).

There were no genome-wide significant findings in the global meta-analysis of prioritised SNPs across all discovery and replication samples (Table S4). Global meta-analysis results with the greatest evidence of association (p≤0.001) are displayed in Table 2. We created forest plots for SNPs with p-value≤0.001 with studies ordered by increasing age (Figure S3). Our study had 90% power at the genome-wide significance level (p = 5×10^−8^) to detect SNPs that explain 0.5% of the genetic variance in men (total n = 10,345), 0.4% in women (total n = 12,031) and 0.2% in the combined set (total n = 22,376).

**Table 2:** Global meta-analysis results with p≤0.001.

STRATUM	SNP	GENE	EA	EAF	BETA	SE	P	I^2^	N
**WOMEN (age adjusted)**	rs13097456	*BDH1*	T	0.304	0.152	0.043	**4.21E-04**	0.479	12108
**WOMEN (age adjusted)**	rs9997081	*LEF1* (37 kb away)	T	0.133	−0.195	0.061	**0.001**	0.348	12094
**WOMEN (age & BMI adjusted)**	rs17665125	*C2orf43, GDF7* (93 kb away)	T	0.723	−0.084	0.025	**9.24E-04**	0.559	12079
**MEN (age adjusted)**	rs11908586	*KCNG1* (130 kb away), *NFATC2* (239 kb away)	G	0.848	−0.422	0.111	**1.48E-04**	0.099	10219
**MEN (age & BMI adjusted)**	rs7176881	*EIF2AK4*	T	0.153	−0.221	0.057	**1.07E-04**	0.430	10203
**COMBINED (age & BMI adjusted)**	rs1476587	*DMTF1* (85 kb away), *GRM3* (203 kb away)	G	0.141	0.283	0.063	**6.50E-06**	0.321	20674

EA - effect allele; EAF - effect allele frequency; SE - standard error; P - p-value; I^2^- measure of heterogeneity; N - total number of samples.

We compared the effect sizes of the initially prioritised 31 SNPs between sexes in the discovery dataset. The effect sizes of loci arising from the combined analyses across men and women are comparable. However, we found heterogeneity in the effect estimates between sexes for the loci prioritized on the basis of the male-only or female-only analyses (Table S5).

The rs9939609 *FTO* SNP showed nominal evidence for association in the age adjusted strata in men and in the combined set across both sexes (Table 3). The adjustment for BMI almost totally eliminates the effect of *FTO* on BC which is visible through a ten-fold decrease of beta values (Table 3). If the *FTO* BC effect were of the same magnitude as the rs9939609 *FTO* SNP effect on BMI [33] our study would have >90% power at the genome-wide significance level to detect it in the combined set, 57% power in women and 40% power in men. We additionally examined 53 previously associated obesity (BMI, waist to hip ratio, weight) SNPs with BC and found 20 SNPs showing p<0.05, across six different strata in the discovery panel (binomial p = 0.175). The most associated SNP was rs6548238 from the *TMEM18* gene with p = 8.15×10^−4^ in the age adjusted stratum across both sexes. Association analysis results for all 53 SNPs are presented in Table S6.

**Table 3:** Global meta-analysis results for the rs9939609 *FTO* SNP.

STRATUM	EA	EAF	BETA	SE	P	I^2^	N
**WOMEN (age adjusted)**	T	0.565	−0.052	0.035	0.137	0.552	11600
**WOMEN (age & BMI adjusted)**	T	0.565	−0.006	0.021	0.769	0.120	11493
**MEN (age adjusted)**	T	0.575	−0.113	0.054	0.036	0.545	9734
**MEN (age & BMI adjusted)**	T	0.575	−0.014	0.033	0.656	0.413	9719
**COMBINED (age adjusted)**	T	0.570	−0.073	0.029	0.013	0.605	21334
**COMBINED (age & BMI adjusted)**	T	0.570	−0.011	0.019	0.571	0.321	21306

EA - effect allele; EAF - effect allele frequency; SE - standard error; P - p-value; I^2^- measure of heterogeneity; N - total number of samples.

We examined the association of 64 previously established T2D loci with BC in our discovery cohort and found 25 SNPs showing p<0.05, across six different strata in the discovery panel (binomial p = 0.11) (Table S7). We also examined 41 previously established SNPs that influence glycaemic traits (fasting glucose, fasting insulin, glycated haemoglobin, 2 h glucose test) and found 12 SNPs with p<0.05, across six different strata in the discovery panel (binomial p = 0.576) (Table S8).

## Discussion

In this large-scale GWAS meta-analysis of BC we analysed a total of 22,376 individuals (12,031 women and 10,345 men) of European ancestry across 14 GWAS discovery datasets and 4 replication cohorts. We followed-up signals from various strata in a two stage replication effort but found no signals reaching genome-wide significance.

Our study had 90% power to detect SNPs that explain 0.2% of the genetic variance in the combined set i.e. our study is well powered to detect modest effects at common loci at the genome-wide significance level (e.g. a risk allele with frequency 0.35 and per-allele increase of 2 mm of BC). One of the weaknesses of this study is that BC is not a clearly-characterised phenotype because it is essentially a composite of muscle mass, skeletal size and fat tissue, traits with distribution differences between sexes [1], [2]. In order to decrease potential misclassification of the phenotype and to search for sex-specific BC effects we conducted analyses in men and women separately. We also analysed the more powerful, combined dataset in the search for shared loci underlying BC. Our study focused on common variants only and has not examined the effect of low frequency and rare variants on BC.

All association analyses were adjusted for age since BC varies throughout the life of individuals. In young individuals BC may be particularly different since it may reflect body development and growth during adolescence. To avoid associations that may arise because BC in adolescents might not be comparable with the phenotypic measure of middle-aged and elder individuals, we performed a sensitivity analysis by excluding studies with adolescent participants. The results of our sensitivity analysis were qualitatively similar to the results from the full-set discovery meta-analysis (data not shown).

We have also examined the effects of top loci across all strata in the discovery dataset in order to compare effect sizes between sexes (Table S5). As expected, the effect sizes of loci prioritized based on the combined male and female analyses are comparable between men and women. However, when we examined the effect estimates of loci prioritised on the basis of a specific sex analysis in the opposite sex, we mainly observe heterogeneity i.e. effect sizes from one sex analyses greatly differ in size or direction in the other sex. This is corroborated with statistical evidence for heterogeneity as evaluated through the I^2^ measure. This may explain why the combined analyses of these loci did not result in improved or genome-wide significance. This additionally confirms that the BC measure has different properties in men and women.

One of the aims of this study was to investigate if established obesity and other fat distribution-associated loci also regulate peripheral adiposity. We expected to see some overlap, since BC is correlated with BMI (Table 1), but also to potentially detect new loci that may explain the properties of peripheral adiposity since fat is disproportionally distributed over the body. We examined the well-established BMI-associated *FTO* gene variant [33], [34] in our dataset consisting of 21,414 individuals and did not find strong evidence for association with BC. On the basis of anthropometric and animal model research it is shown that the *FTO* gene is primarily associated with fat mass [34], [35]. This may explain the fact that we observe only nominal association with BC, since BC is a composite measure of, both lean and fat mass. Of the 53 previously established obesity/adiposity loci we examined in the discovery dataset, only one had p<0.001 (rs6548238 SNP in the *TMEM18*, p = 8.15×10^−4^ in the combined-age adjusted set). Variants in the *TMEM18* gene were previously associated with BMI, body weight and increased risk of childhood obesity [33], [34], [36]. Willer et al showed that associations of the *TMEM18* gene with BMI are, similarly as for the *FTO* gene, driven by increased fat mass [33].

We have also investigated if established T2D loci and loci implicated in glycaemic traits are associated with BC. We did not detect strong evidence for association with BC. Only one previously established T2D variant (rs11899863 in the *THADA* gene) had p = 0.001 in the men age-and-BMI adjusted set. This gene was previously associated with thyroid adenomas [37] and recently with gestational weight gain [38].

Within the power constraints of our study, the data suggest that BC is not a good surrogate measure for overall adiposity genetics. To get a better measure of muscularity we employed BMI adjustments for a subset of our analyses, assuming that BMI is an indirect measure of overall adiposity. This interpretation may not be straightforward because BMI is stature-dependent and may reflect lean tissue as well [13], [34]. No genome-wide replicating associations were identified.

In this large-scale GWAS meta-analysis for BC we have not identified any signals reaching genome-wide significance and do not observe robust association of previously established obesity loci with BC. This may indicate that peripheral adiposity, measured through BC, does not share similar biological patterns with overall adiposity, measured through BMI, and that BC is not a good measure of peripheral adiposity but mainly reflects muscularity. We have also carried out six stratified analyses, which although non-independent give rise to multiplicity issues. Any significant or nominal associations would therefore need to be interpreted with added caution. Large-scale collaborative efforts will be required to achieve the necessary power to detect loci underpinning BC, and detailed anthropometry will help deconvolute the determinants of muscularity, adiposity and their distributions.

## Supporting Information

Figure S1 **A bar chart of BC measures across studies.**
(PDF)

Figure S2 **Manhattan and QQ plots based on meta-analyses results of the discovery panel:** a) women – age adjusted, b) women – age and BMI adjusted, c) men - age adjusted, d) men – age and BMI adjusted.
(PDF)

Figure S3 **Forest plots for global meta-analysis SNPs with p-value≤0.001 with studies ordered by increasing age (the top study contains the youngest individuals).** This comparison can indicate the presence of age effects on associations with BC. We have not found clear evidence of age effects on BC. Box areas are proportional to study sample size.
(PDF)

Table S1 **Detailed sample characteristics on men, women and combined set.**
(PDF)

Table S2 **Study-specific information on genotyping platform, imputation method and QC metrics.**
(PDF)

Table S3 **The number of directly genotyped and imputed meta-analysed SNPs.**
(PDF)

Table S4 **Discovery dataset, replication stage 1 and 2 and global-meta-analysis results of prioritised SNPs.** CHR - chromosome; POS - position; EA - effect allele; NEA - non-effect allele; EAF - effect allele frequency; SE - standard error; P - p-value; I^2^- measure of heterogeneity; N - total number of samples (22 stage 2 replication SNPs are shown in bold).
(PDF)

Table S5 **Comparison of effect sizes of top results between sexes in the discovery dataset.** CHR - chromosome; POS - position; EA - effect allele; NEA - non-effect allele; EAF - effect allele frequency; SE - standard error; P - p-value; I^2^- measure of heterogeneity; N - total number of samples.
(PDF)

Table S6 **Association of established obesity SNPs with BC.** CHR - chromosome; POS - position; EA - effect allele; NEA - non-effect allele; EAF - effect allele frequency; SE- standard error; P - p-value; I^2^- measure of heterogeneity; N - total number of samples (p-values<0.05 in bold).
(PDF)

Table S7 **Association of established T2D loci.** CHR - chromosome; POS - position; EA - effect allele; NEA - non-effect allele; EAF - effect allele frequency; SE- standard error; P - p-value; I^2^- measure of heterogeneity; N - total number of samples (p-values<0.05 in bold).* SNP rs7564886 is a proxy for originally associated T2D SNP rs7578597 (r2 = 1).
(PDF)

Table S8 **Association of loci influencing glycemic traits.** CHR - chromosome; POS - position; EA - effect allele; NEA - non-effect allele; EAF - effect allele frequency; SE- standard error; P - p-value; I^2^- measure of heterogeneity; N - total number of samples (p-values<0.05 in bold);FGlu - fasting glucose; FIns - fasting insulin; 2 hrGlu - 2 h after glucose challenge; HbA1C - glycated hemoglobin.
(PDF)
