# Supplementary figures and images for: Genome-Wide Association Study to Identify Common Variants Associated with Brachial Circumference: A Meta-Analysis of 14 Cohorts
Source: PLoS One. 2012 Mar 29;7(3):e31369. doi: 10.1371/journal.pone.0031369 (PMC3315559; doi:10.1371/journal.pone.0031369)

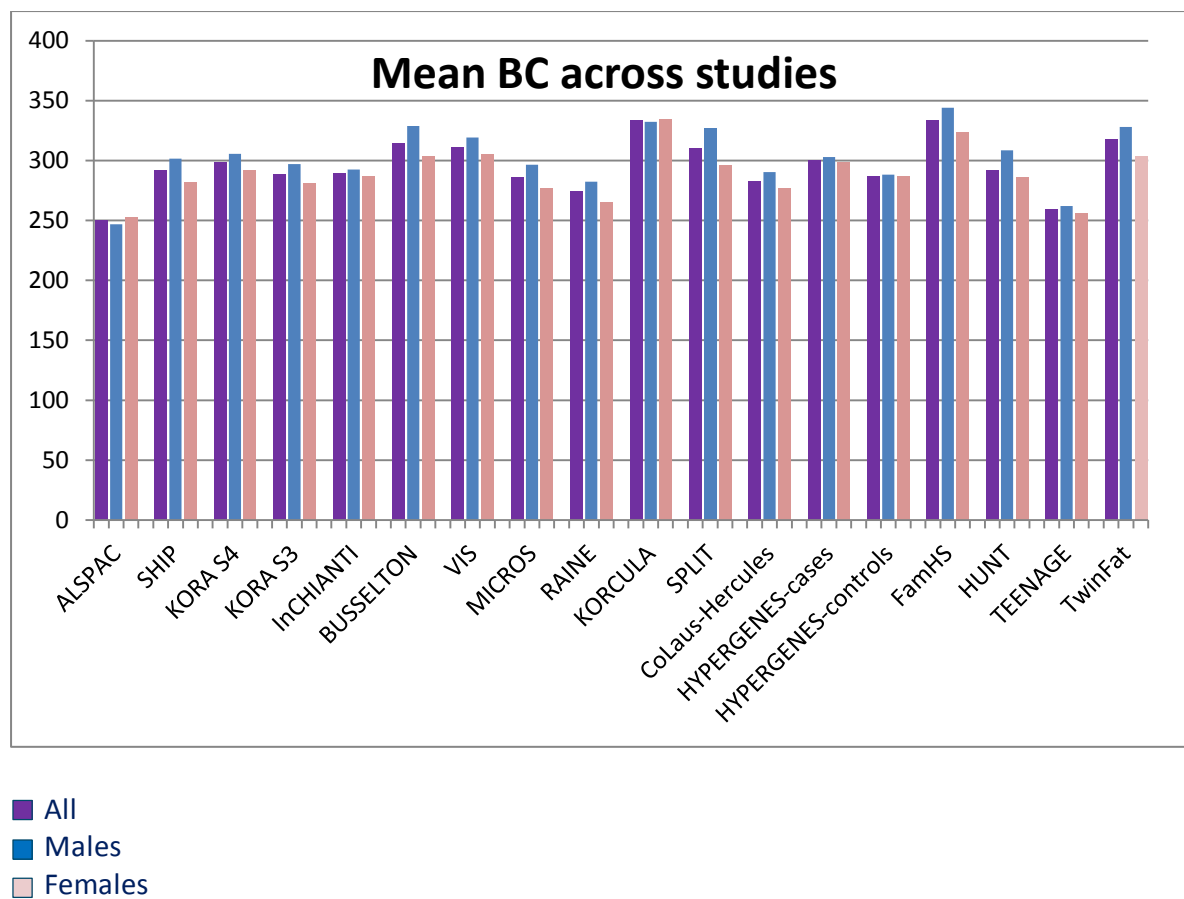

Figure S1. A bar chart of BC measures across studies.

Supplement: Figure S1 — A bar chart of BC measures across studies. (PDF) [file pone.0031369.s001.pdf]
