# Supplementary material for: Genome-Wide Association Study to Identify Common Variants Associated with Brachial Circumference: A Meta-Analysis of 14 Cohorts
Source: PLoS One. 2012 Mar 29;7(3):e31369. doi: 10.1371/journal.pone.0031369 (PMC3315559; doi:10.1371/journal.pone.0031369)
Supplement: Figure S2 — Manhattan and QQ plots based on meta-analyses results of the discovery panel: a) women – age adjusted, b) women – age and BMI adjusted, c) men - age adjusted, d) men – age and BMI adjusted. (PDF) [file pone.0031369.s002.pdf]

a)

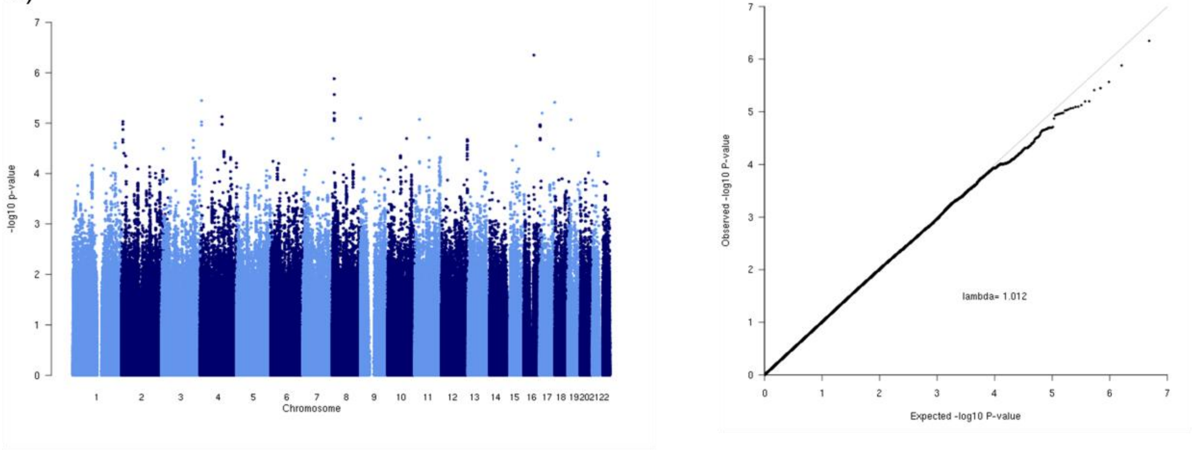
-log10 p-value
Chromosome
1 2 3 4 5 6 7 8 9 10 11 12 13 14 15 16 17 18 19 20 21 22
Observed -log10 P-value
Expected -log10 P-value
lambda= 1.012

b)

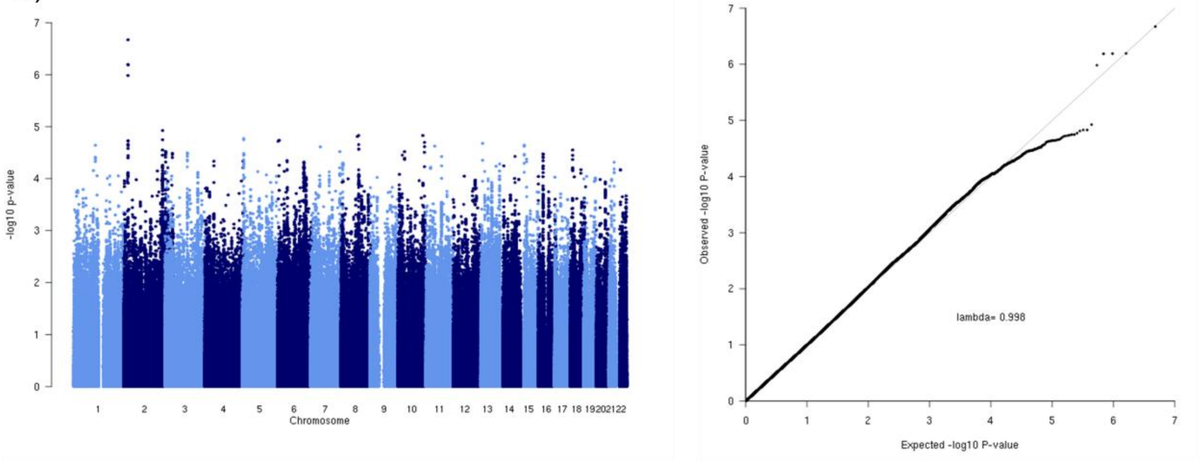
-log10 p-value
Chromosome
1 2 3 4 5 6 7 8 9 10 11 12 13 14 15 16 17 18 19 20 21 22
Observed -log10 P-value
Expected -log10 P-value
lambda= 0.998

c)

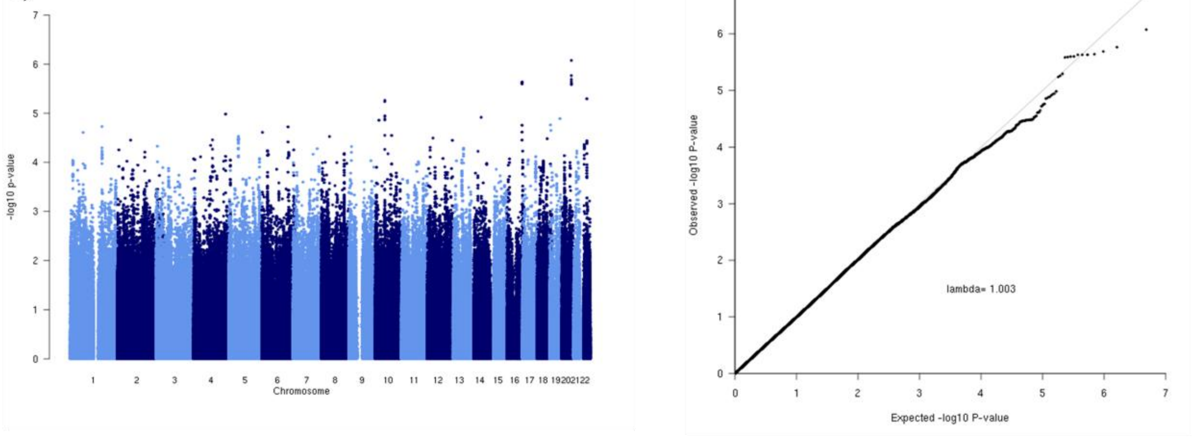
-log10 p-value
Chromosome
1 2 3 4 5 6 7 8 9 10 11 12 13 14 15 16 17 18 19 20 21 22
Observed -log10 P-value
Expected -log10 P-value
lambda= 1.003

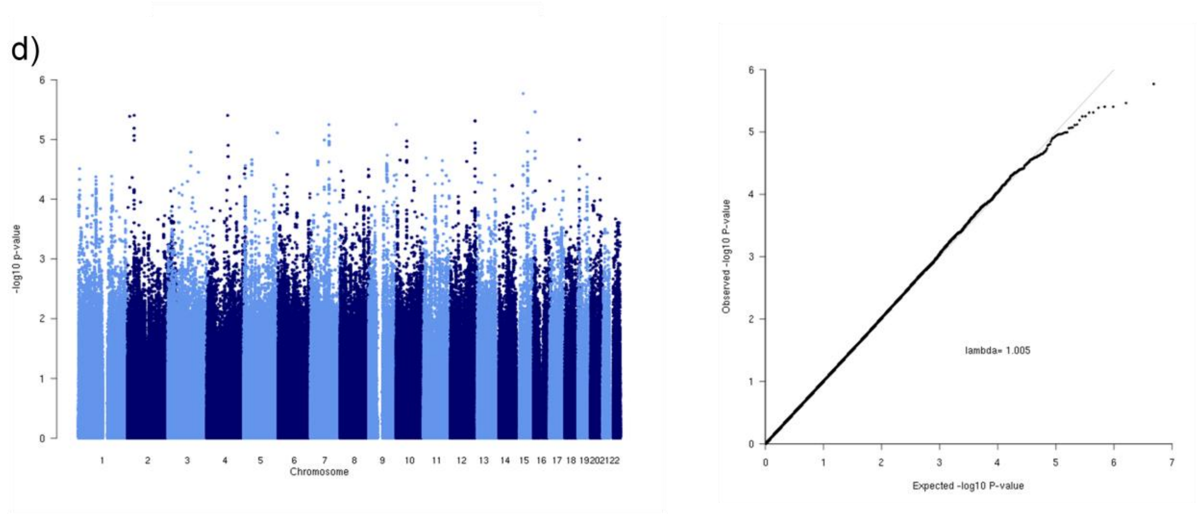

Figure S2. Manhattan and QQ plots based on meta-analyses results of the discovery panel: a) women – age adjusted, b) women – age and BMI adjusted, c) men - age adjusted, d) men – age and BMI adjusted.
