# Supplementary material for: Genome-Wide Association Study to Identify Common Variants Associated with Brachial Circumference: A Meta-Analysis of 14 Cohorts
Source: PLoS One. 2012 Mar 29;7(3):e31369. doi: 10.1371/journal.pone.0031369 (PMC3315559; doi:10.1371/journal.pone.0031369)
Supplement: Figure S3 — Forest plots for global meta-analysis SNPs with p-value≤0.001 with studies ordered by increasing age (the top study contains the youngest individuals). This comparison can indicate the presence of age effects on associations with BC. We have not found clear evidence of age effects on BC. Box areas are proportional to study sample size. (PDF) [file pone.0031369.s003.pdf]

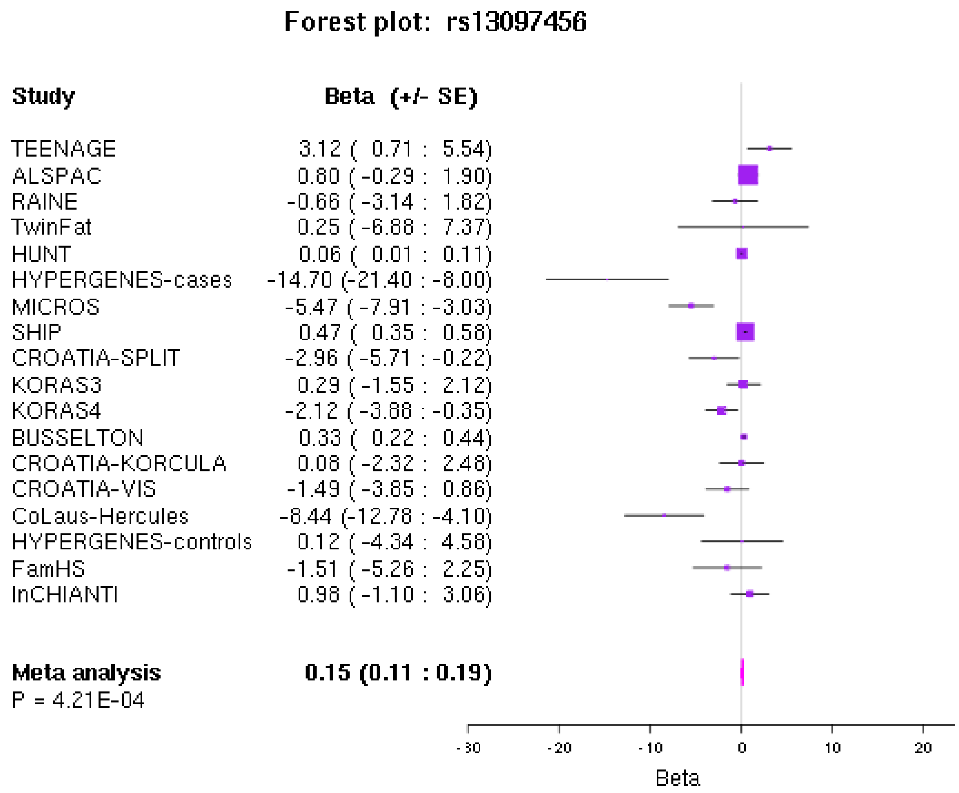
Forest plot: rs13097456
Study
Beta (+/- SE)
TEENAGE 3.12 (0.71 : 5.54)
ALSPAC 0.80 (-0.29 : 1.90)
RAINE -0.66 (-3.14 : 1.82)
TwinFat 0.25 (-6.88 : 7.37)
HUNT 0.06 (0.01 : 0.11)
HYPERGENES-cases -14.70 (-21.40 : -8.00)
MICROS -5.47 (-7.91 : -3.03)
SHIP 0.47 (0.35 : 0.58)
CROATIA-SPLIT -2.96 (-5.71 : -0.22)
KORAS3 0.29 (-1.55 : 2.12)
KORAS4 -2.12 (-3.88 : -0.35)
BUSSELTON 0.33 (0.22 : 0.44)
CROATIA-KORCULA 0.08 (-2.32 : 2.48)
CROATIA-VIS -1.49 (-3.85 : 0.86)
CoLaus-Hercules -8.44 (-12.78 : -4.10)
HYPERGENES-controls 0.12 (-4.34 : 4.58)
FamHS -1.51 (-5.26 : 2.25)
InCHIANTI 0.98 (-1.10 : 3.06)
Meta analysis 0.15 (0.11 : 0.19)
P = 4.21E-04
-30
-20
-10
0
10
20
Beta

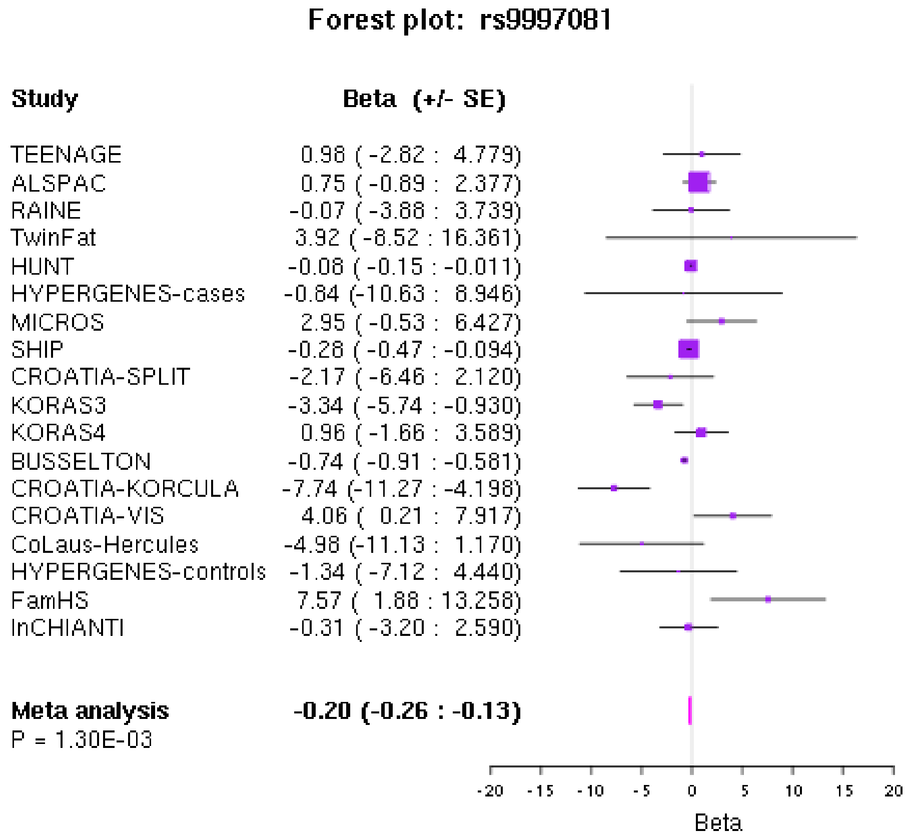
Forest plot: rs9997081
Study
Beta (+/- SE)
TEENAGE 0.98 (-2.82 : 4.779)
ALSPAC 0.75 (-0.89 : 2.377)
RAINE -0.07 (-3.88 : 3.739)
TwinFat 3.92 (-8.52 : 16.361)
HUNT -0.08 (-0.15 : -0.011)
HYPERGENES-cases -0.84 (-10.63 : 8.946)
MICROS 2.95 (-0.53 : 6.427)
SHIP -0.28 (-0.47 : -0.094)
CROATIA-SPLIT -2.17 (-6.46 : 2.120)
KORAS3 -3.34 (-5.74 : -0.930)
KORAS4 0.96 (-1.66 : 3.589)
BUSSELTON -0.74 (-0.91 : -0.581)
CROATIA-KORCULA -7.74 (-11.27 : -4.198)
CROATIA-VIS 4.06 (0.21 : 7.917)
CoLaus-Hercules -4.98 (-11.13 : 1.170)
HYPERGENES-controls -1.34 (-7.12 : 4.440)
FamHS 7.57 (1.88 : 13.258)
InCHIANTI -0.31 (-3.20 : 2.590)
Meta analysis -0.20 (-0.26 : -0.13)
P = 1.30E-03
-20
-15
-10
-5
0
5
10
15
20
Beta

Forest plot: rs17665125

Study	Beta (+/- SE)
TEENAGE	0.68 (-0.725 : 2.083)
ALSPAC	-1.27 (-1.755 : -0.791)
RAINE	-2.43 (-3.845 : -1.011)
TwinFat	1.77 (-1.765 : 5.309)
HUNT	-0.04 (-0.071 : -0.017)
HYPERGENES-cases	-2.64 (-7.500 : 2.220)
MICROS	-0.98 (-2.960 : 1.008)
SHIP	-0.36 (-0.438 : -0.284)
CROATIA-SPLIT	1.32 (-0.296 : 2.931)
KORAS3	0.12 (-1.034 : 1.264)
KORAS4	-0.95 (-2.142 : 0.246)
BUSSELTON	1.53 (0.245 : 2.811)
CROATIA-KORCULA	-1.26 (-2.894 : 0.376)
CROATIA-VIS	-1.79 (-3.506 : -0.065)
CoLaus-Hercules	-3.68 (-5.900 : -1.460)
HYPERGENES-controls	-0.39 (-3.580 : 2.800)
FamHS	-4.35 (-6.227 : -2.479)
InCHIANTI	-2.35 (-4.250 : -0.454)
**Meta analysis**	**-0.08 (-0.11 : -0.059)**

P = 9.24E-04

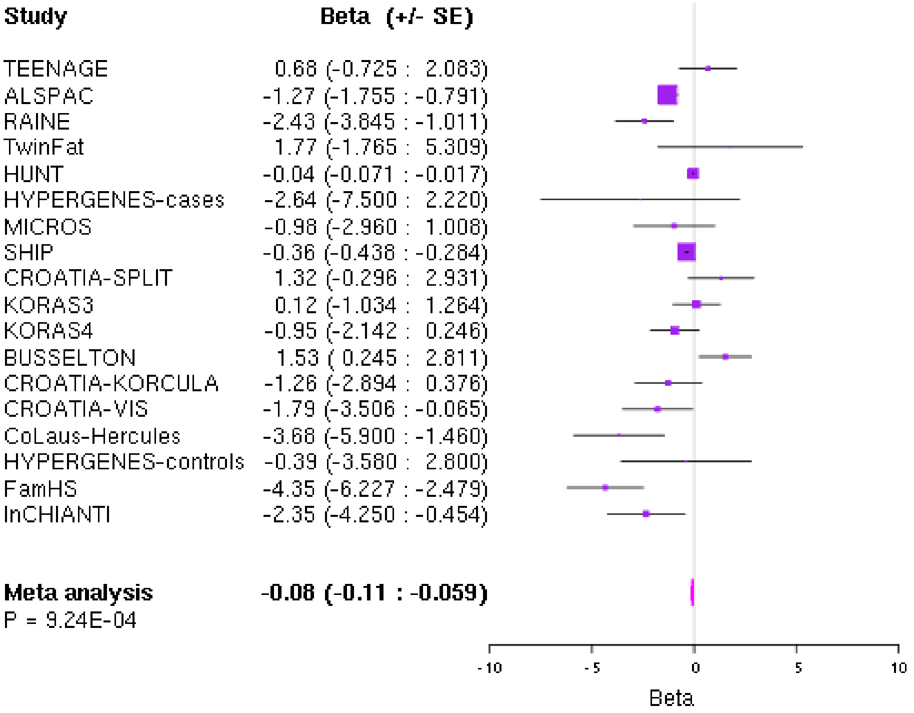

Forest plot: rs11908586

Study	Beta (+/- SE)
TEENAGE	6.00 (1.49 : 10.520)
ALSPAC	-2.74 (-4.68 : -0.796)
RAINE	-3.52 (-7.90 : 0.865)
TwinFat	-8.14 (-16.60 : 0.320)
HYPERGENES-cases	-8.63 (-17.35 : 0.090)
HUNT	-0.07 (-0.22 : 0.091)
MICROS	-0.48 (-4.31 : 3.342)
CROATIA-SPLIT	-4.04 (-9.22 : 1.136)
SHIP	-0.75 (-0.91 : -0.588)
KORAS3	-2.35 (-4.58 : -0.109)
KORAS4	0.23 (-1.94 : 2.399)
BUSSELTON	-2.46 (-7.23 : 2.303)
CROATIA-VIS	1.12 (-3.06 : 5.295)
CROATIA-KORCULA	-5.88 (-10.76 : -1.002)
CoLaus-Hercules	-1.41 (-5.49 : 2.670)
HYPERGENES-controls	0.89 (-6.95 : 8.715)
FamHS	-1.71 (-5.93 : 2.515)
InCHIANTI	-3.31 (-6.47 : -0.154)
**Meta analysis**	**-0.42 (-0.53 : -0.31)**

P = 1.48E-04

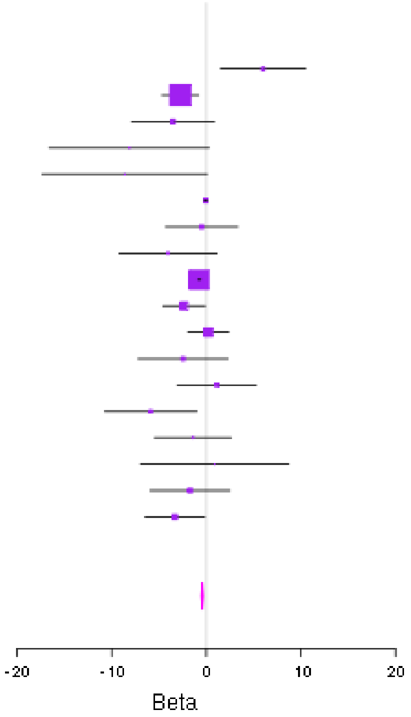

Forest plot: rs7176881

Study	Beta (+/- SE)
TEENAGE	-0.78 (-2.16 : 0.601)
ALSPAC	-1.06 (-1.71 : -0.414)
RAINE	-1.19 (-3.24 : 0.862)
TwinFat	9.25 (4.51 : 13.982)
HYPERGENES-cases	8.26 (3.51 : 13.010)
HUNT	-0.08 (-0.15 : -0.002)
MICROS	-2.66 (-4.63 : -0.689)
CROATIA-SPLIT	-0.94 (-3.92 : 2.039)
SHIP	-0.40 (-0.49 : -0.306)
KORAS3	-1.95 (-3.10 : -0.798)
KORAS4	-0.96 (-2.20 : 0.268)
BUSSELTON	-1.04 (-3.03 : 0.947)
CROATIA-VIS	-1.72 (-4.10 : 0.666)
CROATIA-KORCULA	-7.49 (-10.87 : -4.122)
CoLaus-Hercules	1.30 (-1.22 : 3.820)
HYPERGENES-controls	4.34 (1.17 : 7.510)
FamHS	-0.37 (-2.54 : 1.795)
InCHIANTI	-2.15 (-3.96 : -0.335)
**Meta analysis**	**-0.22 (-0.28 : -0.16)**

P = 1.07E-04

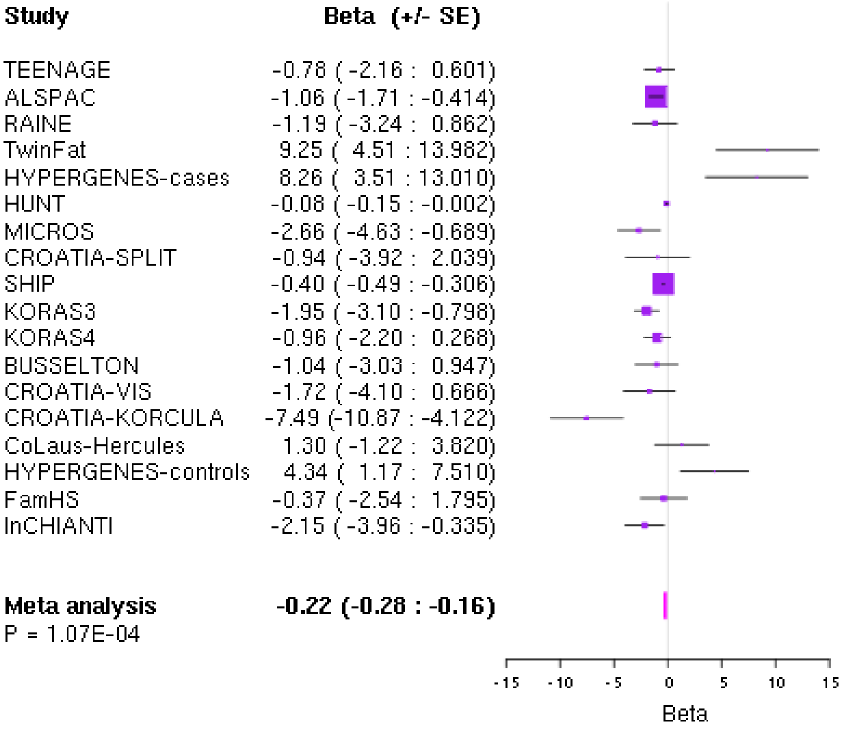

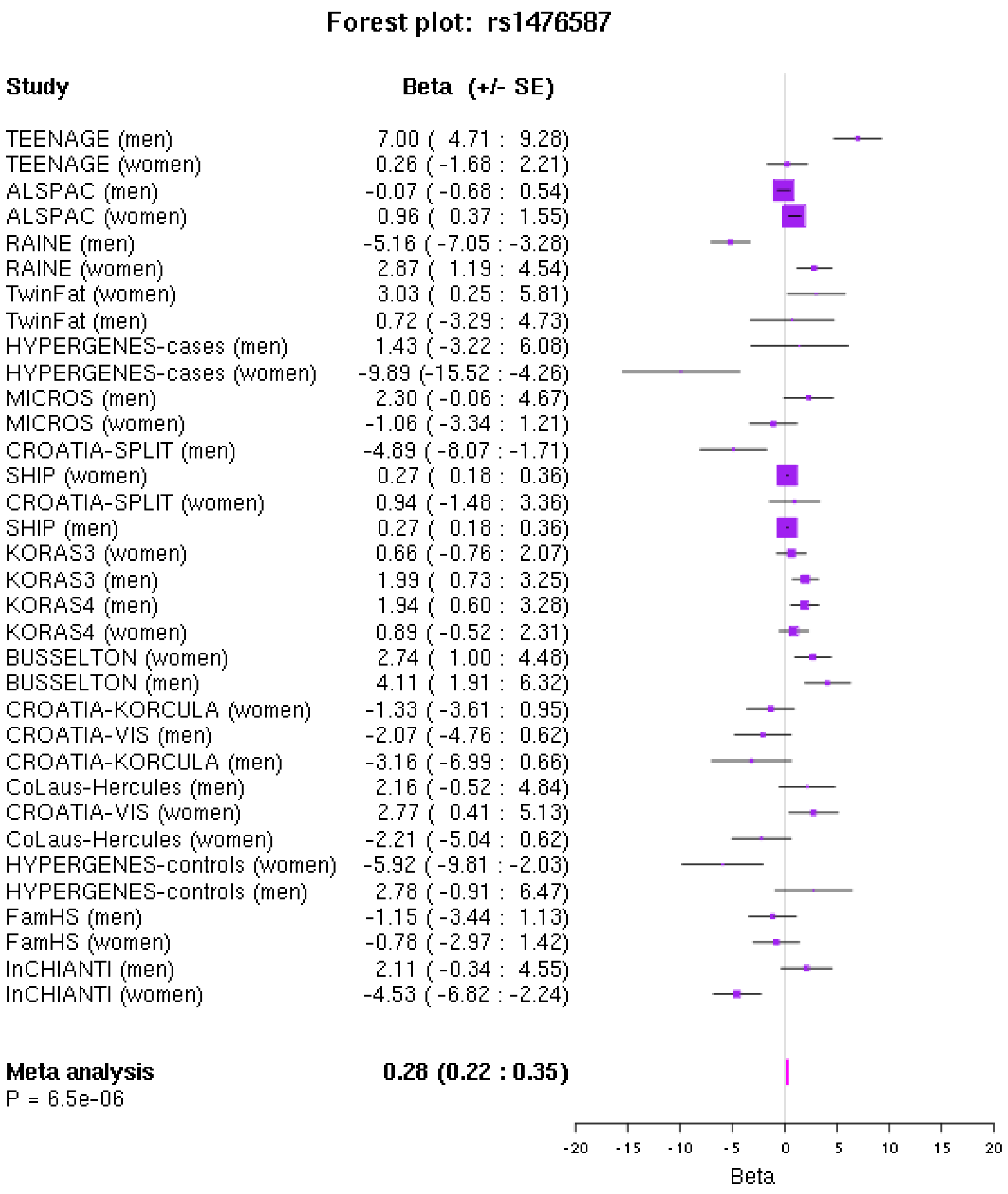

Figure S3. Forest plots for global meta-analysis SNPs with p-value ≤ 0.001 with studies ordered by increasing age (the top study contains the youngest individuals). This comparison can indicate the presence of age effects on associations with BC. We have not found clear evidence of age effects on BC.

Box areas are proportional to study sample size.
