# Supplementary material for: Genome-Wide Association Study to Identify Common Variants Associated with Brachial Circumference: A Meta-Analysis of 14 Cohorts
Source: PLoS One. 2012 Mar 29;7(3):e31369. doi: 10.1371/journal.pone.0031369 (PMC3315559; doi:10.1371/journal.pone.0031369)
Supplement: Table S1 — Detailed sample characteristics on men, women and combined set. (PDF) [file pone.0031369.s004.pdf]

Table S1. Detailed sample characteristics on men, women and combined set

Cohort	Sample (n)	Age, years Mean (stdev)	BC, mm Mean (stdev)	BMI, kg/m^2 Mean (stdev)	Correlation BC and BMI
**DISCOVERY DATASET**					
	all (4428)	13.77(0.21)	250.03 (31.93)	20.22 (3.25)	0.9162
	boys (2161)	13.77 (0.21)	246.89 (32.12)	19.83 (3.15)	0.9178
**ALSPAC**	girls (2267)	13.78 (0.21)	253.05 (31.47)	20.60 (3.31)	0.9143
	all (4070)	49.73 (16.27)	291.61 (33.17)	27.31 (4.77)	0.759
	men (2004)	50.88 (16.43)	301.68 (28.40)	27.68 (4.04)	0.697
**SHIP**	women (2066)	48.60 (16.04)	281.84 (34.53)	26.95 (5.35)	0.82
	all (1788)	53.78 (8.89)	298.79 (29.87)	27.60 (4.30)	0.764
	men (871)	54.04 (8.94)	305.62 (24.84)	27.88 (3.60)	0.71
**KORA S4**	women (917)	53.53 (8.83)	292.29 (32.68)	27.34 (4.85)	0.804
	all (1634)	52.65 (10.08)	288.96 (27.33)	27.28 (4.03)	0.762
	men (811)	53.10 (10.09)	296.90 (23.05)	27.69 (3.44)	0.704
**KORA S3**	women (823)	52.20 (10.07)	281.14 (28.93)	26.88 (4.50)	0.809
	all (1169)	68.09 (15.38)	289.54 (32.67)	27.19 (4.15)	0.681
	men (522)	67.08 (15.42)	292.38 (30.56)	27.04 (3.37)	0.653
**InCHIANTI**	women (647)	68.91 (15.31)	287.24 (34.14)	27.30 (4.68)	0.709
	all (924)	54.15 (17.19)	314.51 (36.62)	25.97 (4.08)	0.842
	men (394)	54.20 (17.12)	328.70 (30.95)	26.77 (3.56)	0.765
**BUSSELTON**	women (530)	54.12 (17.24)	303.97 (36.97)	25.38 (4.34)	0.887
	all (905)	56.27 (15.52)	311.12 (34.22)	27.28 (4.18)	0.775
	men (384)	55.93(14.98)	319.12 (30.99)	27.49 (3.69)	0.726
**CROATIA-VIS**	women (521)	56.53(15.94)	305.23 (35.31)	27.14 (4.51)	0.815
	all (895)	44.99 (16.82)	285.70 (32.84)	25.34 (4.64)	0.721
	men (491)	44.85 (16.16)	296.49 (29.64)	25.82 (4.01)	0.668
**MICROS**	women (404)	45.11 (17.33)	276.69 (32.70)	24.97 (5.05)	0.765
	all (884)	17.03 (0.23)	274.2 (34.91)	23.07 (4.37)	0.841
	boys (454)	17.01 (0.21)	282.4 (35.29)	22.98 (4.41)	0.819
**RAINE**	girls (430)	17.06 (0.25)	265.5 (32.34)	23.17 (4.32)	0.862
	all (841)	56.28 (13.98)	333.72 (47.20)	27.91 (4.19)	0.592
	men (304)	55.94 (13.75)	332.24 (41.06)	27.76 (4.09)	0.684
**CROATIA-KORCULA**	women (537)	55.50 (13.8)	334.57 (50.44)	27.99 (4.39)	0.553
	all (495)	49.04 (14.65)	310.30 (36.26)	26.93 (4.19)	0.857
	men (210)	47.85 (15.55)	326.81 (28.61)	27.9 (3.47)	0.796
**CROATIA-SPLIT**	women (285)	49.84 (13.95)	296.35(32.76)	25.97 (4.2)	0.899
	all (369)	56.93 (10.31)	283.19 (29.41)	25.89 (4.15)	0.82
	men (193)	56.50 (10.44)	290.41 (23.89)	26.70 (3.29)	0.71
**CoLaus-Hercules**	women (176)	57.32 (10.21)	276.61 (32.34)	25.14 (4.69)	0.86
	all (155)	41.02 (8.77)	300.8 (35.3)	27.38 (5.16)	0.77
	men (73)	40.71 (9.05)	303.0 (28.5)	26.78 (3.89)	0.63
**HYPERGENES-cases**	women (82)	41.29 (8.56)	298.8 (40.5)	27.89 (6.01)	0.84
	all (196)	59.16 (7.62)	287.4 (27.0)	25.93 (3.42)	0.78
	men (89)	59.91 (8.10)	288.3 (25.9)	26.33 (3.46)	0.78
**HYPERGENES-controls**	women (107)	58.53 (7.17)	286.6 (28.0)	25.60 (3.37)	0.79
**REPLICATION STAGE 1**					
	all (967)	62.5 (11.3)	333.5 (43.5)	29 (5.3)	0.806
	men (466)	60 (12.3)	344.1 (37)	29.6 (4.7)	0.737
**FamHS (*in silico*)**	women (501)	64.8 (9.7)	323.8 (47.2)	28.5 (6)	0.853
**REPLICATION STAGE 2**					
	all (1626)	40.9 (12.19)	292.2 (30.12)	26.0 (4.09)	0.81
	men (432)	42.1 (11.81)	308.6 (25.76)	26.5 (3.59)	0.79
**HUNT (*in silico*)**	women (1194)	40.4 (12.31)	286.2 (29.39)	25.8 (4.24)	0.85
	all (819)	13.42 (0.85)	259.03 (33.27)	21.19 (3.45)	0.899
	boys (365)	13.4 (0.85)	262.14 (33.66)	21.27 (3.49)	0.908
**TEENAGE (*de novo*)**	girls (454)	13.43 (0.85)	256.54 (32.78)	21.12 (3.41)	0.893
	all (211)	27.65 (2.13)	317.48 (41.11)	25.15 (4.54)	0.856
	men (121)	27.9 (1.98)	327.93 (38.81)	25.46 (4.08)	0.828
**TwinFat (*in silico*)**	women (90)	27.3 (2.28)	303.43 (40.13)	24.75 (5.09)	0.932
