# Supplementary material for: Genome-Wide Association Study to Identify Common Variants Associated with Brachial Circumference: A Meta-Analysis of 14 Cohorts
Source: PLoS One. 2012 Mar 29;7(3):e31369. doi: 10.1371/journal.pone.0031369 (PMC3315559; doi:10.1371/journal.pone.0031369)
Supplement: Table S2 — Study-specific information on genotyping platform, imputation method and QC metrics. (PDF) [file pone.0031369.s005.pdf]

Table S2. Study-specific information on genotyping platform, imputation method and QC metrics

Cohort	Full name	Genotyping platform	Imputation software	Analysis software
**DISCOVERY DATASET**				
**ALSPAC**	Avon Longitudinal Study of Parents and Children	Illumina HUmanHap550K	MACH	MACH2QTL
**SHIP**	Study of Health in Pomerania	Affymetrix 6.0	Impute	QUICKTEST
**KORA S4**	Cooperative Health Research in the Region of Augsburg	Affymetrix 6.0	Impute	SNPTEST
**KORA S3**	Cooperative Health Research in the Region of Augsburg	Affymetrix 500K	Impute	SNPTEST
**InCHIANTI**	Invecchiare nel Chianti	Illumina HUmanHap550K	MACH	MACH2DAT
**BUSSELTON**	Busselton Health Study	Illumina 610-Quad	MACH	MACH2QTL
**CROATIA-VIS**	CROATIA-Vis	Illumina HumanHap300v1	MACH	ProbABEL
**MICROS**	MICROS	Illumina HumHap300v2	MACH	ProbABEL
**RAINE**	Western Australian Pregnancy (Raine) Cohort	Illumina Human660W-Quad	MACH	MACH2DAT
**CROATIA-KORCULA**	CROATIA-Korcula	Illumina HumanHapCNV370	MACH	ProbABEL
**CROATIA-SPLIT**	CROATIA-Split	Illumina HumanHapCNV370	MACH	ProbABEL
**CoLaus-Hercules**	Cohorte Lausannoise	Affymetrix 500K	Impute	Matlab
**HYPERGENES-controls**	HYPERGENES-controls	Illumina HumanHap 1M Duo	MACH	Matlab
**HYPERGENES-cases**	HYPERGENES-cases	Illumina HumanHap 1M Duo	MACH	Matlab
**REPLICATION STAGE 1**				
**FamHS**	Family Heart Study	Illumina HumanHap550K	MACH	SAS MIXED
**REPLICATION STAGE 2**				
**HUNT**	Nord-Trøndelag Health Study	Illumina Human670-Quad	Impute	SNPTEST
**TwinFat**	TwinFat	Illumina Human670-Quad	Impute	PLINK

Cohort	SNP call rate	HWE treshold	Number of analysed SNPs, men	Number of analysed SNPs, women	PMID number/Reference
**DISCOVERY DATASET**					
**ALSPAC**	97	< 5x10-7	2167058	2167058	11237119
**SHIP**	NA	NA	2213047	2213011	20167617
**KORA F4**	none	none	2220986	2220032	16032514
**KORA F3**	none	none	2081533	2081289	16032514
**InCHIANTI**	98	< 1x10-4	2170095	2170095	11129752, 18464913
**BUSSELTON**	95	< 1x10-7	2169082	2169082	/
**CROATIA-VIS**	98	< 1x10-6	2140384	2142890	8327257
**MICROS**	98	< 1x10-10	2135716	2137627	17550581
**RAINE**	95	< 1x10-6	2177749	2177749	8105165, 9224128, 8855394
**CROATIA-KORCULA**	98	< 1x10-6	2134339	2139632	19260141
**CROATIA-SPLIT**	98	< 1x10-6	2147731	2150855	/
**COLAUS**	70	< 1x10-7	2088926	2088334	17701901, 19543373, 18366642
**HYPERGENES-controls**	90	< 1x10-7	2163460	2158028	20935630
**HYPERGENES-cases**	90	< 1x10-7	2144072	2142993	20935630
**REPLICATION STAGE 1**					
**FamHS**	98	< 1x10-6	31	31	8651220
**REPLICATION STAGE 2**					
**HUNT**	99	< 1x10-6	23	23	Holmen et al. 2003
**TwinFat**	95	< 1x10-6	23	23	17254406, 19584879
