# Supplementary material for: Genome-Wide Association Study to Identify Common Variants Associated with Brachial Circumference: A Meta-Analysis of 14 Cohorts
Source: PLoS One. 2012 Mar 29;7(3):e31369. doi: 10.1371/journal.pone.0031369 (PMC3315559; doi:10.1371/journal.pone.0031369)
Supplement: Table S3 — The number of directly genotyped and imputed meta-analysed SNPs. (PDF) [file pone.0031369.s006.pdf]

Table S3. The number of directly genotyped and imputed meta-analysed SNPs

**Meta-analysis**	**No analysed SNPs**	**GC**
WOMEN (age adjusted)	2413846	1.012
WOMEN (age & BMI adjusted)	2413164	0.998
MEN (age adjusted)	2421371	1.003
MEN (age & BMI adjusted)	2423014	1.005
COMBINED (age adjusted)	2437372	1.01
COMBINED (age & BMI adjusted)	2438430	1.017

GC - genomic control
