# Supplementary material for: Genome-Wide Association Study to Identify Common Variants Associated with Brachial Circumference: A Meta-Analysis of 14 Cohorts
Source: PLoS One. 2012 Mar 29;7(3):e31369. doi: 10.1371/journal.pone.0031369 (PMC3315559; doi:10.1371/journal.pone.0031369)
Supplement: Table S4 — Discovery dataset, replication stage 1 and 2 and global-meta-analysis results of prioritised SNPs. CHR - chromosome; POS - position; EA - effect allele; NEA - non-effect allele; EAF - effect allele frequency; SE - standard error; P - p-value; I2- measure of heterogeneity; N - total number of samples (22 stage 2 replication SNPs are shown in bold). (PDF) [file pone.0031369.s007.pdf]

Table S4. Discovery dataset, replication stage 1 and 2 and global-meta-analysis results of prioritised SNPs

**DISCOVERY DATASET**											**REPLICATION STAGE 1 (FamHS)**						
**WOMEN (age adjusted)**																	
**CHR**	**POS**	**MARKER**	**EA**	**NEA**	**EAF**	**BETA**	**SE**	**P**	**I^2**	**N**	**EA**	**NEA**	**EAF**	**BETA**	**SE**	**N**	**P**
2	6783934	rs13413734	G	A	0.057	-1.010	0.228	9.30E-06	0	7119	A	G	0.951	-2.481	6.974	501	0.722
3	198756854	**rs13097456**	T	A	0.313	0.371	0.080	3.57E-06	0.345	9893	A	T	0.737	1.507	3.755	501	0.689
4	109346002	**rs9997081**	T	A	0.141	-0.548	0.122	7.46E-06	0	9875	T	A	0.086	7.569	5.689	501	0.185
8	10201669	**rs4240644**	G	A	0.175	-0.464	0.096	1.32E-06	0	9892	A	G	0.873	2.386	4.407	501	0.589
**WOMEN (age & BMI adjusted)**																	
2	20809243	rs4971547	G	A	0.764	-0.359	0.072	6.42E-07	0	9880	A	G	0.216	4.516	1.793	501	**0.013**
2	20826151	rs17721572	T	C	0.236	0.363	0.073	6.47E-07	0.033	9880	T	C	0.217	4.528	1.817	501	**0.014**
2	20827120	**rs17665125**	T	A	0.773	-0.392	0.075	2.13E-07	0.049	9880	A	T	0.202	4.353	1.874	501	**0.021**
**MEN (age adjusted)**																	
10	49728569	rs11101533	T	C	0.265	0.493	0.109	5.76E-06	0	8041	C	T	0.739	-3.166	2.322	466	0.175
16	77910813	rs8063785	T	C	0.878	-0.757	0.161	2.53E-06	0	8852	T	C	0.92	-1.534	4.498	466	0.734
16	77910834	rs16950229	T	C	0.123	0.760	0.161	2.34E-06	0	8852	C	T	0.92	-1.534	4.497	466	0.733
16	77911400	**rs8043805**	G	A	0.123	0.760	0.161	2.33E-06	0	8851	A	G	0.92	-1.535	4.495	466	0.733
20	49202621	**rs11908586**	G	A	0.879	-0.784	0.159	8.42E-07	0	8852	G	A	0.928	-1.709	4.224	466	0.686
20	49207656	rs6021011	G	A	0.887	-0.776	0.162	1.72E-06	0	8852	G	A	0.937	-3.031	4.16	466	0.467
22	32498478	**rs4821182**	T	C	0.409	0.410	0.090	5.08E-06	0.601	8848	T	C	0.412	0.908	2.312	466	0.695
**MEN (age & BMI adjusted)**																	
2	33065018	**rs219145**	G	C	0.659	0.267	0.058	3.94E-06	0	8791		/	/	/	/	/	/
7	91390466	rs10243083	G	A	0.508	-0.256	0.058	8.47E-06	0.313	8838	G	A	0.495	0.932	1.531	466	0.544
12	122198883	**rs1727302**	G	A	0.280	0.296	0.065	4.88E-06	0	8837	A	G	0.747	-0.082	1.726	466	0.962
15	38054128	**rs7176881**	T	C	0.160	-0.434	0.091	1.70E-06	0.184	8837	C	T	0.897	0.373	2.168	466	0.863
15	59691743	**rs7178929**	G	A	0.214	0.327	0.073	7.62E-06	0.022	8838	A	G	0.811	-1.72	1.845	466	0.354
15	96627749	**rs11638366**	T	C	0.424	0.271	0.058	3.44E-06	0	8838	T	C	0.442	2.75	1.54	466	0.076
**COMBINED (age adjusted)**																	
4	72824274	rs17383291	T	G	0.866	-0.536	0.109	8.50E-07	0	18339	G	T	0.088	3.221	4.962	966	0.517
4	119641561	**rs4833582**	T	C	0.455	0.249	0.054	3.94E-06	0.329	18745	T	C	0.452	1.65	1.995	966	0.409
**COMBINED (age & BMI adjusted)**																	
3	107243063	**rs1478786**	T	C	0.177	0.270	0.059	4.51E-06	0	18716	C	T	0.863	1.615	1.665	966	0.332
3	107245050	**rs2399060**	T	C	0.825	-0.265	0.058	5.95E-06	0	18646	T	C	0.863	1.634	1.664	966	0.327
3	158795136	**rs9845279**	G	C	0.469	0.241	0.054	8.63E-06	0	18349	C	G	0.515	4.151	1.4	966	**0.003**
4	105192645	**rs13133212**	G	C	0.819	-0.288	0.065	8.19E-06	0.229	16714	G	C	0.879	-0.812	2.176	966	0.709
7	86529190	**rs13243613**	T	C	0.145	0.282	0.063	8.93E-06	0.324	18718	C	T	0.891	1.256	1.538	966	0.414
7	86534650	**rs1476587**	G	A	0.145	0.282	0.063	8.92E-06	0.325	18718	A	G	0.891	1.256	1.538	966	0.414
8	14510052	**rs7837164**	T	C	0.794	-0.244	0.055	8.34E-06	0.157	18582	T	C	0.824	2.215	1.405	966	0.116
8	86322348	**rs2132589**	G	A	0.657	-0.211	0.044	1.42E-06	0.416	18611	G	A	0.66	-0.107	1.28	966	0.933
8	86327248	**rs10090196**	T	C	0.335	0.209	0.044	1.63E-06	0.398	18717	C	T	0.666	-0.122	1.283	966	0.924
8	86369541	rs16913721	G	A	0.648	-0.204	0.043	2.15E-06	0.342	18716	G	A	0.648	-0.07	1.217	966	0.954

CHR - chromosome; POS - position; EA - effect allele; NEA - non-effect allele; EAF - effect allele frequency; SE - standard error; P - p-value; I^2- measure of heterogeneity; N - total number of samples (22 stage 2 replication SNPs are shown in bold)

DISCOVERY AND REPLICATION STAGE 1									REPLICATION STAGE 2							
**WOMEN (age adjusted)**																
**MARKER**	**EA**	**NEA**	**EAF**	**BETA**	**SE**	**P**	**I^2**	**N**	**EA**	**NEA**	**EAF**	**BETA**	**SE**	**P**	**I^2**	**N**
rs13413734	G	A	0.056	-1.008	0.228	9.61E-06	0	7620	NA	NA	NA	NA	NA	NA	NA	NA
**rs13097456**	T	A	0.311	0.371	0.080	3.74E-06	0.304	10394	T	A	0.263	0.062	0.051	0.221	0	1714
**rs9997081**	T	A	0.138	-0.544	0.122	8.56E-06	0.0413	10376	T	A	0.100	-0.081	0.070	0.250	0	1718
**rs4240644**	G	A	0.173	-0.465	0.096	1.25E-06	0	10393	G	A	0.137	-0.014	0.061	0.813	0	1716
**WOMEN (age & BMI adjusted)**																
rs4971547	G	A	0.765	-0.366	0.072	3.87E-07	0.228	10381	NA	NA	NA	NA	NA	NA	NA	NA
rs17721572	T	C	0.235	0.370	0.073	3.93E-07	0.251	10381	NA	NA	NA	NA	NA	NA	NA	NA
**rs17665125**	T	A	0.774	-0.398	0.075	1.32E-07	0.228	10381	T	A	0.408	-0.044	0.027	0.106	0	1698
**MEN (age adjusted)**																
rs11101533	T	C	0.264	0.498	0.109	4.45E-06	0	8507	NA	NA	NA	NA	NA	NA	NA	NA
rs8063785	T	C	0.880	-0.758	0.161	2.42E-06	0	9318	NA	NA	NA	NA	NA	NA	NA	NA
rs16950229	T	C	0.120	0.760	0.161	2.24E-06	0	9318	NA	NA	NA	NA	NA	NA	NA	NA
**rs8043805**	G	A	0.121	0.761	0.161	2.23E-06	0	9317	G	A	0.084	-0.044	0.131	0.734	0	901
**rs11908586**	G	A	0.882	-0.786	0.159	7.96E-07	0	9318	G	A	0.501	-0.061	0.157	0.695	0.264182	901
rs6021011	G	A	0.889	-0.780	0.162	1.54E-06	0	9318	NA	NA	NA	NA	NA	NA	NA	NA
**rs4821182**	T	C	0.409	0.410	0.090	4.82E-06	0.571	9314	T	C	0.396	-0.028	0.069	0.688	0	900
**MEN (age & BMI adjusted)**																
**rs219145**	not typed								G	C	0.368	-0.011	0.045	0.808	0.336	869
rs10243083	G	A	0.508	-0.255	0.057	9.56E-06	0.283	9304	NA	NA	NA	NA	NA	NA	NA	NA
**rs1727302**	G	A	0.278	0.296	0.065	4.90E-06	0	9303	G	A	0.288	-0.102	0.047	**0.030**	0	901
**rs7176881**	T	C	0.157	-0.434	0.091	1.68E-06	0.121	9303	T	C	0.114	-0.076	0.074	0.305	0.516526	900
**rs7178929**	G	A	0.213	0.329	0.073	6.51E-06	0	9304	G	A	0.164	-0.066	0.057	0.249	0.451668	902
**rs11638366**	T	C	0.425	0.274	0.058	2.51E-06	0	9304	T	C	0.403	-0.008	0.041	0.849	0	894
**COMBINED (age adjusted)**																
rs17383291	T	G	0.868	-0.536	0.109	8.17E-07	0	19305	NA	NA	NA	NA	NA	NA	NA	NA
**rs4833582**	T	C	0.455	0.250	0.054	3.71E-06	0.309	19711	T	C	0.478	-0.065	0.036	0.072	0	2614
**COMBINED (age & BMI adjusted)**																
**rs1478786**	T	C	0.175	0.269	0.059	4.97E-06	0	19682	T	C	0.126	0.005	0.031	0.862	0	2613
**rs2399060**	T	C	0.827	-0.263	0.058	6.55E-06	0	19612	T	C	0.415	-0.005	0.031	0.865	0	2615
**rs9845279**	G	C	0.470	0.238	0.054	1.16E-05	0.054	19315	G	C	0.486	-0.007	0.027	0.805	0.584392	2463
**rs13133212**	G	C	0.822	-0.288	0.065	8.00E-06	0.194	17680	G	C	0.400	0.009	0.031	0.776	0	2599
**rs13243613**	T	C	0.143	0.281	0.063	9.67E-06	0.307	19684	T	C	0.130	-0.022	0.029	0.450	0.493532	2619
**rs1476587**	G	A	0.143	0.280	0.063	9.81E-06	0.309	19684	G	A	0.090	2.154	1.309	0.100	0	990
**rs7837164**	T	C	0.796	-0.242	0.055	9.56E-06	0.162	19548	T	C	0.399	0.001	0.029	0.963	0.261923	2621
**rs2132589**	G	A	0.658	-0.210	0.044	1.42E-06	0.394	19577	G	A	0.480	-0.005	0.022	0.804	0.382487	2598
**rs10090196**	T	C	0.335	0.209	0.044	1.63E-06	0.374	19683	T	C	0.318	0.001	0.022	0.955	0	2613
rs16913721	G	A	0.648	-0.204	0.043	2.16E-06	0.316	19682	NA	NA	NA	NA	NA	NA	NA	NA

GLOBAL META-ANALYSIS								
**WOMEN (age adjusted)**								
**MARKER**	**EA**	**NEA**	**EAF**	**BETA**	**SE**	**P**	**I^2**	**N**
rs13413734	G	A	0.056	-1.008	0.228	9.61E-06	0	7620
**rs13097456**	T	A	0.304	0.152	0.043	**4.21E-04**	0.479	12108
**rs9997081**	T	A	0.133	-0.195	0.061	**0.001**	0.348	12094
**rs4240644**	G	A	0.168	-0.145	0.051	0.005	0.342	12109
**WOMEN (age & BMI adjusted)**								
rs4971547	G	A	0.765	-0.366	0.072	3.87E-07	0.228	10381
rs17721572	T	C	0.235	0.370	0.073	3.93E-07	0.251	10381
**rs17665125**	T	A	0.723	-0.084	0.025	**9.24E-04**	0.559	12079
**MEN (age adjusted)**								
rs11101533	T	C	0.264	0.498	0.109	4.45E-06	0	8507
rs8063785	T	C	0.880	-0.758	0.161	2.42E-06	0	9318
rs16950229	T	C	0.120	0.760	0.161	2.24E-06	0	9318
**rs8043805**	G	A	0.117	0.281	0.101	0.006	0.374	10218
**rs11908586**	G	A	0.848	-0.422	0.111	**1.48E-04**	0.099	10219
rs6021011	G	A	0.889	-0.780	0.162	1.54E-06	0	9318
**rs4821182**	T	C	0.408	0.137	0.054	0.012	0.660	10214
**MEN (age & BMI adjusted)**								
**rs219145**	G	C	0.633	0.096	0.035	0.007	0.474	9660
rs10243083	G	A	0.508	-0.255	0.057	9.56E-06	0.283	9304
**rs1727302**	G	A	0.279	0.037	0.038	0.325	0.530	10204
**rs7176881**	T	C	0.153	-0.221	0.057	**1.07E-04**	0.430	10203
**rs7178929**	G	A	0.208	0.086	0.045	0.054	0.528	10206
**rs11638366**	T	C	0.423	0.087	0.033	0.009	0.399	10198
**COMBINED (age adjusted)**								
rs17383291	T	G	0.868	-0.536	0.109	8.17E-07	0	19305
**rs4833582**	T	C	0.458	0.033	0.030	0.269	0.532	22325
**COMBINED (age & BMI adjusted)**								
**rs1478786**	T	C	0.169	0.063	0.027	0.021	0.205	22295
**rs2399060**	T	C	0.779	-0.062	0.027	0.022	0.205	22227
**rs9845279**	G	C	0.471	0.042	0.024	0.082	0.463	21778
**rs13133212**	G	C	0.768	-0.048	0.028	0.086	0.444	20279
**rs13243613**	T	C	0.142	0.031	0.026	0.240	0.523	22303
**rs1476587**	G	A	0.141	0.283	0.063	**6.50E-06**	0.321	20674
**rs7837164**	T	C	0.749	-0.053	0.026	0.040	0.431	22169
**rs2132589**	G	A	0.637	-0.047	0.020	0.016	0.546	22175
**rs10090196**	T	C	0.333	0.044	0.020	0.025	0.523	22296
rs16913721	G	A	0.648	-0.204	0.043	2.16E-06	0.316	19682
