# Supplementary material for: Genome-Wide Association Study to Identify Common Variants Associated with Brachial Circumference: A Meta-Analysis of 14 Cohorts
Source: PLoS One. 2012 Mar 29;7(3):e31369. doi: 10.1371/journal.pone.0031369 (PMC3315559; doi:10.1371/journal.pone.0031369)
Supplement: Table S5 — Comparison of effect sizes of top results between sexes in the discovery dataset. CHR - chromosome; POS - position; EA - effect allele; NEA - non-effect allele; EAF - effect allele frequency; SE - standard error; P - p-value; I2- measure of heterogeneity; N - total number of samples. (PDF) [file pone.0031369.s008.pdf]

Table S5. Comparison of effect sizes of top results between sexes in the discovery dataset.

STRATUM FROM WHICH SNPs WERE PRIORITISED FOR FOLLOW-UP											RESULTS ACROSS OTHER STRATA									
CHR	POS	MARKER	EA	NEA	EAF	BETA	SE	P	I^2	N	BETA	SE	P	I^2	N	BETA	SE	P	I^2	N
WOMEN (age adjusted)											MEN (age adjusted)					COMBINED (age adjusted)				
2	6783934	rs13413734	G	A	0.057	-1.010	0.228	9.30E-06	0	7119	0.345	0.197	0.080	0.035	6940	-0.238	0.149	0.111	0.572	14059
3	198756854	rs13097456	T	A	0.313	0.371	0.080	3.57E-06	0.345	9893	-0.082	0.099	0.409	0.034	8851	0.192	0.062	0.002	0.434	18744
4	109346002	rs9997081	T	A	0.141	-0.548	0.122	7.46E-06	0	9875	0.150	0.164	0.361	0	8835	-0.299	0.098	0.002	0.251	18710
8	10201669	rs4240644	G	A	0.175	-0.464	0.096	1.32E-06	0	9892	0.038	0.123	0.757	0	8851	-0.274	0.076	2.93E-04	0.017	18743
WOMEN (age & BMI adjusted)											MEN (age & BMI adjusted)					COMBINED (age & BMI adjusted)				
2	20809243	rs4971547	G	A	0.764	-0.359	0.072	6.42E-07	0	9880	-0.059	0.069	0.391	0.178	8838	-0.202	0.050	4.90E-05	0.303	18718
2	20826151	rs17721572	T	C	0.236	0.363	0.073	6.47E-07	0.033	9880	0.065	0.070	0.349	0.164	8838	0.207	0.050	3.84E-05	0.306	18718
2	20827120	rs17665125	T	A	0.773	-0.392	0.075	2.13E-07	0.049	9880	-0.059	0.072	0.413	0	8838	-0.219	0.052	2.89E-05	0.275	18718
MEN (age adjusted)											WOMEN (age adjusted)					COMBINED (age adjusted)				
10	49728569	rs11101533	T	C	0.265	0.493	0.109	5.76E-06	0	8041	-0.076	0.080	0.344	0	9069	0.125	0.065	0.053	0.324	17110
16	77910813	rs8063785	T	C	0.878	-0.757	0.161	2.53E-06	0	8852	0.069	0.124	0.578	0	9893	-0.240	0.098	0.015	0.300	18745
16	77910834	rs16950229	T	C	0.123	0.760	0.161	2.34E-06	0	8852	-0.068	0.124	0.582	0	9892	0.241	0.098	0.014	0.285	18744
16	77911400	rs8043805	G	A	0.123	0.760	0.161	2.33E-06	0	8851	-0.072	0.124	0.563	0	9893	0.239	0.098	0.015	0.286	18744
20	49202621	rs11908586	G	A	0.879	-0.784	0.159	8.42E-07	0	8852	-0.070	0.130	0.591	0	9893	-0.355	0.101	4.21E-04	0	18745
20	49207656	rs6021011	G	A	0.887	-0.776	0.162	1.72E-06	0	8852	-0.065	0.131	0.621	0	9607	-0.346	0.102	7.01E-04	0.006	18459
22	32498478	rs4821182	T	C	0.409	0.410	0.090	5.08E-06	0.601	8848	0.055	0.067	0.418	0.198	9888	0.183	0.054	6.73E-04	0.529	18736
MEN (age & BMI adjusted)											WOMEN (age & BMI adjusted)					COMBINED (age & BMI adjusted)				
2	33065018	rs219145	G	C	0.659	0.267	0.058	3.94E-06	0	8791	0.065	0.063	0.304	0	9850	0.174	0.043	4.32E-05	0	18641
7	91390466	rs10243083	G	A	0.508	-0.256	0.058	8.47E-06	0.313	8838	0.071	0.059	0.228	0	9879	-0.096	0.041	0.019	0.423	18717
12	122198883	rs1727302	G	A	0.280	0.296	0.065	4.88E-06	0	8837	0.009	0.069	0.902	0.022	9880	0.161	0.047	6.57E-04	0.192	18717
15	38054128	rs7176881	T	C	0.160	-0.434	0.091	1.70E-06	0.184	8837	0.049	0.092	0.596	0.015	9879	-0.195	0.065	0.002	0.385	18716
15	59691743	rs7178929	G	A	0.214	0.327	0.073	7.62E-06	0.022	8838	0.070	0.079	0.375	0.381	9880	0.208	0.054	1.04E-04	0.302	18718
15	96627749	rs11638366	T	C	0.424	0.271	0.058	3.44E-06	0	8838	0.023	0.061	0.709	0.110	9880	0.152	0.042	2.86E-04	0.202	18718
COMBINED (age adjusted)											WOMEN (age adjusted)					MEN (age adjusted)				
4	72824274	rs17383291	T	G	0.866	-0.536	0.109	8.50E-07	0	18339	-0.487	0.137	0.0004	0	9892	-0.616	0.178	5.55E-04	0	8447
4	119641561	rs4833582	T	C	0.455	0.249	0.054	3.94E-06	0.329	18745	0.202	0.068	0.003	0.541	9893	0.331	0.089	2.11E-04	0	8852
COMBINED (age & BMI adjusted)											WOMEN (age & BMI adjusted)					MEN (age & BMI adjusted)				
3	107243063	rs1478786	T	C	0.177	0.270	0.059	4.51E-06	0	18716	0.340	0.083	4.37E-05	0	9879	0.201	0.083	0.016	0	8837
3	107245050	rs2399060	T	C	0.825	-0.265	0.058	5.95E-06	0	18646	-0.337	0.083	4.58E-05	0	9842	-0.193	0.083	0.019	0	8804
3	158795136	rs9845279	G	C	0.469	0.241	0.054	8.63E-06	0	18349	0.229	0.078	0.003	0	9687	0.254	0.075	7.48E-04	0	8662
4	105192645	rs13133212	G	C	0.819	-0.288	0.065	8.19E-06	0.229	16714	-0.182	0.093	0.051	0	8864	-0.384	0.090	1.93E-05	0.492	7850
7	86529190	rs13243613	T	C	0.145	0.282	0.063	8.93E-06	0.324	18718	0.288	0.091	0.002	0.295	9880	0.275	0.088	0.002	0.395	8838
7	86534650	rs1476587	G	A	0.145	0.282	0.063	8.92E-06	0.325	18718	0.288	0.091	0.002	0.296	9880	0.275	0.088	0.002	0.395	8838
8	14510052	rs7837164	T	C	0.794	-0.244	0.055	8.34E-06	0.157	18582	-0.176	0.078	0.025	0.011	9810	-0.310	0.076	4.87E-05	0.210	8772
8	86322348	rs2132589	G	A	0.657	-0.211	0.044	1.42E-06	0.416	18611	-0.277	0.064	1.48E-05	0.366	9826	-0.154	0.060	0.010	0.422	8785
8	86327248	rs10090196	T	C	0.335	0.209	0.044	1.63E-06	0.398	18717	0.270	0.064	2.21E-05	0.325	9879	0.156	0.060	0.009	0.426	8838
8	86369541	rs16913721	G	A	0.648	-0.204	0.043	2.15E-06	0.342	18716	-0.245	0.063	1.05E-04	0.247	9879	-0.169	0.059	0.004	0.405	8837

CHR - chromosome; POS - position; EA - effect allele; NEA - non-effect allele; EAF - effect allele frequency; SE - standard error; P - p-value; I^2- measure of heterogeneity; N - total number of samples
