# Supplementary material for: Genome-Wide Association Study to Identify Common Variants Associated with Brachial Circumference: A Meta-Analysis of 14 Cohorts
Source: PLoS One. 2012 Mar 29;7(3):e31369. doi: 10.1371/journal.pone.0031369 (PMC3315559; doi:10.1371/journal.pone.0031369)
Supplement: Table S6 — Association of established obesity SNPs with BC. CHR - chromosome; POS - position; EA - effect allele; NEA - non-effect allele; EAF - effect allele frequency; SE- standard error; P - p-value; I2- measure of heterogeneity; N - total number of samples (p-values<0.05 in bold). (PDF) [file pone.0031369.s009.pdf]

Table S6. Association of established obesity SNPs with BC

SNP information								
**SNP**	**GENE**	**TRAIT**	**REFERENCE**	**CHR**	**POS**	**EA**	**NEA**	**EAF**
rs2815752	*NEGR1*	BMI	Willer et al. 2009	1	72585028	G	A	0.382
rs1514175	*TNNI3K*	BMI	Speliotes et al. 2010	1	74764232	G	A	0.581
rs1555543	*PTBP2*	BMI	Speliotes et al. 2010	1	96717385	C	A	0.561
rs984222	*TBX15-WARS2*	waist-to-hip	Heid et al. 2010	1	119305366	G	C	0.601
rs1011731	*DNM3-PIGC*	waist-to-hip	Heid et al. 2010	1	170613171	G	A	0.432
rs543874	*SEC16B*	BMI	Speliotes et al. 2010	1	176156103	G	A	0.216
rs10913469	*SEC16B, RASAL2*	BMI/weight	Thorleifsson et al. 2009	1	176180142	T	C	0.782
rs4846567	*LYPLAL1*	waist-to-hip	Heid et al. 2010	1	217817340	T	G	0.292
rs6548238	*TMEM18*	BMI	Willer et al. 2009	2	624905	T	C	0.212
rs713586	*RBJ*	BMI	Speliotes et al. 2010	2	25011512	T	C	0.535
rs887912	*FANCL*	BMI	Speliotes et al. 2010	2	59156381	T	C	0.300
rs2890652	*LRP1B*	BMI	Speliotes et al. 2010	2	142676401	T	C	0.797
rs10195252	*GRB14*	waist-to-hip	Heid et al. 2010	2	165221337	T	C	0.583
rs6784615	*NISCH-STAB1*	waist-to-hip	Heid et al. 2010	3	52481466	T	C	0.869
rs6795735	*ADAMTS9*	waist-to-hip	Heid et al. 2010	3	64680405	T	C	0.445
rs13078807	*CADM2*	BMI	Speliotes et al. 2010	3	85966840	G	A	0.231
rs7647305	*SFRS10, ETV5, DGKG*	BMI/weight	Thorleifsson et al. 2009	3	187316984	T	C	0.241
rs10938397	*GNPDA2*	BMI	Willer et al. 2009	4	44877284	G	A	0.437
rs13107325	*SLC39A8*	BMI	Speliotes et al. 2010	4	103407732	T	C	0.130
rs2112347	*FLJ35779*	BMI	Speliotes et al. 2010	5	75050998	T	G	0.614
rs6235	*PCSK1*	BMI	Benzinou et al. 2008	5	95754654	G	C	0.307
rs4836133	*ZNF608*	BMI	Speliotes et al. 2010	5	124360002	C	A	0.530
rs6861681	*CPEB4*	waist-to-hip	Heid et al. 2010	5	173295064	G	A	0.672
rs1294421	*LY86*	waist-to-hip	Heid et al. 2010	6	6688148	T	G	0.391
rs206936	*NUDT3*	BMI	Speliotes et al. 2010	6	34410847	G	A	0.238
rs6905288	*VEGFA*	waist-to-hip	Heid et al. 2010	6	43866851	G	A	0.452
rs987237	*TFAP2B*	BMI	Speliotes et al. 2010	6	50911009	G	A	0.212
rs9491696	*RSPO3*	waist-to-hip	Heid et al. 2010	6	127494332	G	C	0.477
rs1055144	*NFE2L3*	waist-to-hip	Heid et al. 2010	7	25837634	T	C	0.210
rs10968576	*LRRN6C*	BMI	Speliotes et al. 2010	9	28404339	G	A	0.323
rs10508503	*PTER*	BMI	Meyre et al. 2009	10	16339957	T	C	0.141
rs4929949	*RPL27A*	BMI	Speliotes et al. 2010	11	8561169	T	C	0.510
rs4923461	*BDNF*	BMI/weight	Thorleifsson et al. 2009	11	27613486	G	A	0.248
rs925946	*BDNF*	BMI/weight	Thorleifsson et al. 2009	11	27623778	T	G	0.302
rs10838738	*MTCH2*	BMI	Willer et al. 2009	11	47619625	G	A	0.353
rs718314	*ITPR2-SSPN*	waist-to-hip	Heid et al. 2010	12	26344550	G	A	0.276
rs7138803	*BCDIN3D, FAIM2*	weight	Thorleifsson et al. 2009	12	48533735	G	A	0.604
rs1443512	*HOXC13*	waist-to-hip	Heid et al. 2010	12	52628951	C	A	0.741
rs4771122	*MTIF3*	BMI	Speliotes et al. 2010	13	26918180	G	A	0.262
rs10150332	*NRXN3*	BMI	Speliotes et al. 2010	14	79006717	T	C	0.760
rs11847697	*PRKD1*	BMI	Speliotes et al. 2010	14	29584863	T	C	0.057
rs2241423	*MAP2K5*	BMI	Speliotes et al. 2010	15	65873892	G	A	0.748
rs12444979	*GPRC5B*	BMI	Speliotes et al. 2010	16	19841101	T	C	0.179
rs7498665	*SH2B1, ATP2A1*	BMI	Willer et al. 2009	16	28790742	G	A	0.399
rs1424233	*MAF*	BMI	Meyre et al. 2009	16	78240252	T	C	0.494
rs1805081	*NPC1*	BMI	Meyre et al. 2009	18	19394430	T	C	0.584
rs17782313	*MC4R*	BMI	Willer et al. 2009	18	56002077	T	C	0.733
rs29941	*KCTD15*	BMI	Thorleifsson et al. 2009	19	39001372	G	A	0.668
rs11084753	*KCTD15*	BMI	Willer et al. 2009	19	39013977	G	A	0.640
rs2287019	*QPCTL*	BMI	Speliotes et al. 2010	19	50894012	T	C	0.235
rs3810291	*TMEM160*	BMI	Speliotes et al. 2010	19	52260843	G	A	0.368
rs6013029	*CTNNBL1*	BMI/fat mass	Liu et al. 2008	20	35832994	T	G	0.058
rs4823006	*ZNRF3-KREMEN1*	waist-to-hip	Heid et al. 2010	22	27781671	G	A	0.441

CHR - chromosome; POS - position; EA - effect allele; NEA - non-effect allele; EAF - effect allele frequency; SE- standard error; P - p-value; I^2- measure of heterogeneity; N - total number of samples (p-values<0.05 in bold)

	WOMEN (age adjusted)					WOMEN (age & BMI adjusted)				
**SNP**	**BETA**	**SE**	**P**	**I^2**	**N**	**BETA**	**SE**	**P**	**I^2**	**N**
rs2815752	-0.012	0.069	0.862	0	9881	-0.001	0.063	0.993	0.237	9868
rs1514175	-0.200	0.067	**0.003**	0.03	9861	-0.052	0.060	0.392	0	9848
rs1555543	0.162	0.068	**0.018**	0.318	9891	-0.030	0.061	0.623	0.606	9878
rs984222	-0.005	0.070	0.937	0	9893	0.068	0.061	0.266	0	9880
rs1011731	0.005	0.067	0.939	0	9844	0.122	0.060	**0.044**	0	9831
rs543874	0.165	0.080	**0.040**	0.062	9893	-0.017	0.076	0.825	0	9880
rs10913469	-0.137	0.079	0.082	0.219	9703	0.004	0.076	0.961	0	9695
rs4846567	0.084	0.074	0.255	0	9892	0.032	0.068	0.637	0	9879
rs6548238	-0.213	0.089	**0.017**	0	9822	0.142	0.079	0.073	0.212	9809
rs713586	-0.065	0.067	0.334	0.442	9852	0.078	0.060	0.190	0	9839
rs887912	0.105	0.073	0.151	0.176	9893	-0.052	0.066	0.433	0	9880
rs2890652	-0.140	0.089	0.115	0.061	9892	-0.032	0.077	0.675	0	9879
rs10195252	-0.053	0.069	0.445	0.112	9893	-0.018	0.061	0.766	0	9880
rs6784615	-0.271	0.186	0.145	0	6700	-0.169	0.580	0.771	0	6689
rs6795735	0.125	0.068	0.065	0.269	9696	0.034	0.060	0.573	0.068	9688
rs13078807	0.131	0.084	0.120	0.155	9892	-0.033	0.075	0.658	0.067	9879
rs7647305	-0.106	0.084	0.209	0	9892	0.039	0.073	0.591	0.366	9879
rs10938397	-0.007	0.066	0.916	0.51	9893	0.135	0.060	**0.024**	0.137	9880
rs13107325	0.002	0.124	0.989	0.187	9834	-0.091	0.122	0.455	0.064	9821
rs2112347	0.039	0.069	0.574	0.294	9893	0.019	0.062	0.761	0.234	9880
rs6235	0.029	0.076	0.700	0.205	9892	-0.030	0.068	0.661	0.287	9879
rs4836133	-0.151	0.067	**0.025**	0.349	9246	0.035	0.061	0.568	0.277	9233
rs6861681	0.155	0.076	**0.041**	0.021	9893	0.103	0.066	0.118	0	9880
rs1294421	-0.002	0.069	0.976	0.083	9887	-0.014	0.062	0.817	0	9874
rs206936	0.116	0.086	0.174	0	9892	0.100	0.076	0.190	0	9879
rs6905288	-0.008	0.072	0.907	0	9879	0.027	0.062	0.668	0	9866
rs987237	0.124	0.085	0.147	0.398	9888	-0.048	0.078	0.539	0.289	9875
rs9491696	-0.039	0.067	0.556	0.452	9893	-0.185	0.059	**0.002**	0.697	9880
rs1055144	0.006	0.085	0.940	0.03	9839	0.011	0.079	0.892	0	9826
rs10968576	-0.128	0.071	0.070	0	9893	-0.053	0.065	0.410	0	9880
rs10508503	0.001	0.118	0.992	0	9070	0.016	0.109	0.881	0.281	9057
rs4929949	0.003	0.068	0.968	0.026	9893	0.054	0.061	0.377	0	9880
rs4923461	0.109	0.083	0.186	0.436	9893	0.123	0.071	0.083	0.054	9880
rs925946	-0.058	0.070	0.400	0.133	9892	-0.048	0.065	0.460	0	9879
rs10838738	0.059	0.070	0.401	0	9893	0.022	0.063	0.728	0	9880
rs718314	-0.012	0.080	0.884	0	9892	0.055	0.068	0.416	0.139	9879
rs7138803	0.026	0.067	0.699	0.231	9852	-0.009	0.060	0.885	0.099	9839
rs1443512	0.098	0.079	0.213	0.287	9893	0.056	0.070	0.424	0.323	9880
rs4771122	0.101	0.083	0.220	0	9892	-0.117	0.072	0.106	0	9879
rs10150332	0.015	0.082	0.851	0.577	9893	-0.066	0.072	0.365	0.098	9880
rs11847697	2.133	2.301	0.354	0	2168	0.379	1.337	0.777	0	2168
rs2241423	0.087	0.080	0.275	0.0238	9892	0.088	0.071	0.215	0.435	9879
rs12444979	0.112	0.097	0.244	0.123	9892	0.011	0.084	0.896	0.282	9879
rs7498665	0.051	0.068	0.458	0.022	9893	0.003	0.062	0.962	0	9880
rs1424233	0.108	0.067	0.108	0	9893	-0.014	0.059	0.813	0	9880
rs1805081	-0.010	0.067	0.886	0.309	9893	-0.038	0.059	0.517	0.299	9880
rs17782313	0.024	0.079	0.765	0.339	9889	0.115	0.070	0.100	0	9876
rs29941	0.027	0.072	0.708	0.169	9892	-0.043	0.065	0.506	0.339	9879
rs11084753	0.013	0.073	0.856	0.379	9891	-0.026	0.067	0.702	0.608	9878
rs2287019	0.002	0.097	0.983	0	7960	0.152	0.100	0.129	0.412	7947
rs3810291	-0.018	0.083	0.826	0	9893	-0.075	0.070	0.286	0.023	9880
rs6013029	0.163	0.221	0.462	0	7243	0.006	0.128	0.960	0.237	7233
rs4823006	0.074	0.069	0.285	0	9883	0.036	0.062	0.559	0	9870

	MEN (age adjusted)					MEN (age & BMI adjusted)				
**SNP**	**BETA**	**SE**	**P**	**I^2**	**N**	**BETA**	**SE**	**P**	**I^2**	**N**
rs2815752	-0.134	0.093	0.147	0	8840	0.007	0.059	0.900	0.177	8826
rs1514175	0.021	0.089	0.811	0.234	8823	0.028	0.057	0.630	0.233	8809
rs1555543	-0.071	0.089	0.423	0.233	8851	-0.058	0.057	0.307	0	8837
rs984222	-0.206	0.093	**0.027**	0	8852	-0.100	0.060	0.094	0.223	8838
rs1011731	0.010	0.089	0.907	0	8812	0.006	0.057	0.923	0	8798
rs543874	-0.027	0.116	0.813	0.664	8851	-0.083	0.074	0.264	0	8837
rs10913469	0.030	0.115	0.791	0.699	8690	0.091	0.074	0.215	0	8684
rs4846567	0.050	0.100	0.621	0.053	8851	-0.026	0.064	0.691	0	8837
rs6548238	-0.286	0.119	**0.016**	0.469	8789	-0.022	0.076	0.777	0.43	8775
rs713586	0.098	0.088	0.262	0.272	8812	0.045	0.056	0.427	0.192	8798
rs887912	0.081	0.097	0.406	0	8852	0.060	0.062	0.333	0	8838
rs2890652	-0.240	0.113	**0.033**	0	8852	-0.042	0.072	0.563	0	8838
rs10195252	0.055	0.089	0.538	0.058	8852	0.010	0.057	0.857	0	8838
rs6784615	-0.052	0.193	0.789	0	8183	-0.028	0.124	0.821	0	8646
rs6795735	0.172	0.089	0.052	0.162	8681	0.044	0.057	0.442	0.067	8675
rs13078807	-0.011	0.111	0.918	0	8852	0.113	0.071	0.113	0	8838
rs7647305	-0.111	0.110	0.312	0	8851	0.012	0.070	0.867	0	8837
rs10938397	-0.005	0.089	0.955	0.003	8851	-0.033	0.057	0.559	0	8837
rs13107325	0.237	0.181	0.192	0	8711	0.008	0.115	0.942	0.147	8685
rs2112347	0.194	0.091	**0.033**	0.386	8852	0.049	0.059	0.404	0.222	8838
rs6235	0.138	0.102	0.179	0.127	8852	0.070	0.066	0.289	0	8838
rs4836133	-0.101	0.090	0.265	0.392	8329	-0.091	0.058	0.117	0.064	8315
rs6861681	0.117	0.095	0.220	0	8851	0.041	0.061	0.500	0	8837
rs1294421	-0.007	0.091	0.936	0.183	8843	-0.090	0.058	0.124	0.068	8829
rs206936	0.219	0.115	0.057	0.236	8852	-0.060	0.074	0.416	0.161	8838
rs6905288	0.074	0.093	0.426	0.177	8845	-0.035	0.060	0.555	0.178	8831
rs987237	0.036	0.110	0.742	0.331	8845	-0.047	0.071	0.507	0.039	8831
rs9491696	0.054	0.088	0.536	0.149	8852	-0.037	0.056	0.509	0.372	8838
rs1055144	-0.201	0.114	0.078	0	8802	-0.210	0.073	**0.004**	0	8788
rs10968576	-0.007	0.097	0.942	0	8851	-0.058	0.062	0.350	0.202	8837
rs10508503	-0.105	0.156	0.501	0	7864	-0.095	0.101	0.346	0	7850
rs4929949	-0.012	0.090	0.891	0	8852	0.002	0.057	0.973	0.044	8838
rs4923461	-0.225	0.109	**0.039**	0	8852	-0.129	0.070	0.064	0	8838
rs925946	0.133	0.098	0.174	0.092	8852	0.106	0.063	0.091	0	8838
rs10838738	-0.004	0.094	0.967	0.049	8852	0.029	0.060	0.629	0	8838
rs718314	-0.056	0.099	0.574	0.347	8851	0.002	0.064	0.974	0.251	8837
rs7138803	-0.155	0.089	0.081	0.065	8822	-0.050	0.057	0.375	0.343	8808
rs1443512	0.092	0.107	0.393	0.236	8851	0.041	0.069	0.551	0.138	8837
rs4771122	0.046	0.105	0.664	0.282	8852	0.023	0.067	0.727	0.112	8838
rs10150332	-0.106	0.107	0.319	0.183	8852	-0.168	0.069	**0.014**	0	8838
rs11847697	3.499	2.355	0.137	0	1551	1.016	1.529	0.507	0	1542
rs2241423	0.073	0.107	0.493	0	8851	0.018	0.068	0.797	0	8837
rs12444979	0.028	0.125	0.825	0.0985	8851	-0.037	0.080	0.645	0.231	8837
rs7498665	0.056	0.089	0.531	0	8852	-0.041	0.057	0.471	0.364	8838
rs1424233	-0.101	0.088	0.252	0	8852	-0.062	0.057	0.271	0	8838
rs1805081	0.041	0.088	0.643	0.421	8851	0.022	0.056	0.691	0.063	8837
rs17782313	-0.231	0.102	**0.023**	0.505	8848	-0.026	0.065	0.690	0.014	8834
rs29941	0.233	0.094	**0.014**	0.078	8852	0.102	0.061	0.092	0.318	8838
rs11084753	0.295	0.097	**0.002**	0.304	8848	0.157	0.062	**0.012**	0.141	8834
rs2287019	-0.174	0.151	0.249	0.256	6994	-0.036	0.095	0.709	0	6980
rs3810291	-0.236	0.101	**0.020**	0.0157	8852	-0.114	0.065	0.080	0.401	8838
rs6013029	0.061	0.189	0.748	0	7862	-0.023	0.121	0.850	0	7849
rs4823006	0.090	0.091	0.325	0	8838	0.023	0.058	0.688	0	8824

	COMBINED (age adjusted)					COMBINED (age & BMI adjusted)				
**SNP**	**BETA**	**SE**	**P**	**I^2**	**N**	**BETA**	**SE**	**P**	**I^2**	**N**
rs2815752	-0.055	0.055	0.320	0.011	18721	0.004	0.043	0.926	0.188	18694
rs1514175	-0.120	0.054	**0.026**	0.244	18684	-0.009	0.041	0.821	0	18657
rs1555543	0.076	0.054	0.160	0.343	18742	-0.045	0.042	0.274	0.396	18715
rs984222	-0.078	0.056	0.163	0	18745	-0.019	0.043	0.659	0.041	18718
rs1011731	0.007	0.054	0.899	0	18656	0.061	0.042	0.144	0	18629
rs543874	0.102	0.066	0.124	0.464	18744	-0.049	0.053	0.355	0	18717
rs10913469	-0.083	0.065	0.202	0.519	18393	0.047	0.053	0.371	0	18379
rs4846567	0.072	0.060	0.228	0	18743	0.002	0.047	0.972	0	18716
rs6548238	-0.239	0.071	**8.15E-04**	0.165	18611	0.056	0.055	0.306	0.329	18584
rs713586	-0.004	0.053	0.935	0.372	18664	0.062	0.041	0.133	0.076	18637
rs887912	0.095	0.059	0.105	0	18745	0.008	0.045	0.868	0	18718
rs2890652	-0.179	0.070	**0.010**	0	18744	-0.038	0.053	0.472	0	18717
rs10195252	-0.012	0.055	0.824	0.113	18745	-0.003	0.042	0.934	0	18718
rs6784615	-0.165	0.134	0.217	0	14883	-0.033	0.121	0.784	0	15335
rs6795735	0.143	0.054	**0.008**	0.216	18377	0.038	0.041	0.352	0.062	18363
rs13078807	0.078	0.067	0.245	0	18744	0.043	0.052	0.401	0	18717
rs7647305	-0.107	0.067	0.109	0	18743	0.026	0.051	0.611	0.094	18716
rs10938397	-0.007	0.053	0.898	0.294	18744	0.048	0.041	0.240	0	18717
rs13107325	0.076	0.103	0.461	0.075	18545	-0.039	0.084	0.639	0.116	18506
rs2112347	0.095	0.055	0.083	0.241	18745	0.036	0.043	0.404	0.138	18718
rs6235	0.067	0.061	0.267	0.187	18744	0.022	0.047	0.638	0.093	18717
rs4836133	-0.062	0.054	0.255	0.44	17575	-0.032	0.042	0.452	0.225	17548
rs6861681	0.140	0.060	**0.018**	0	18744	0.068	0.045	0.126	0	18717
rs1294421	-0.004	0.055	0.947	0.114	18730	-0.055	0.042	0.195	0	18703
rs206936	0.153	0.069	**0.026**	0.026	18744	0.017	0.053	0.755	0	18717
rs6905288	0.021	0.057	0.707	0.082	18724	-0.006	0.043	0.886	0	18697
rs987237	0.090	0.067	0.180	0.366	18733	-0.048	0.052	0.363	0.175	18706
rs9491696	-0.005	0.053	0.926	0.34	18745	-0.108	0.041	**0.008**	0.6	18718
rs1055144	-0.066	0.068	0.331	0	18641	-0.110	0.054	**0.042**	0	18614
rs10968576	-0.086	0.057	0.131	0	18744	-0.054	0.045	0.227	0	18717
rs10508503	-0.036	0.094	0.699	0	16934	-0.043	0.074	0.559	0	16907
rs4929949	-0.003	0.054	0.952	0	18745	0.027	0.042	0.514	0	18718
rs4923461	-0.013	0.066	0.848	0.31	18745	-0.005	0.050	0.927	0	18718
rs925946	0.006	0.057	0.910	0.196	18744	0.032	0.045	0.483	0	18717
rs10838738	0.036	0.056	0.523	0	18745	0.026	0.043	0.554	0	18718
rs718314	-0.029	0.062	0.646	0.077	18743	0.026	0.046	0.568	0.208	18716
rs7138803	-0.039	0.053	0.461	0.219	18674	-0.031	0.041	0.458	0.116	18647
rs1443512	0.096	0.063	0.130	0.173	18744	0.049	0.049	0.312	0.151	18717
rs4771122	0.081	0.065	0.213	0	18744	-0.042	0.049	0.393	0.098	18717
rs10150332	-0.030	0.065	0.643	0.456	18745	-0.119	0.050	**0.017**	0	18718
rs11847697	-0.617	1.646	0.708	0.012	3719	0.655	1.007	0.515	0	3710
rs2241423	0.081	0.064	0.203	0	18743	0.052	0.049	0.292	0.167	18716
rs12444979	0.081	0.077	0.291	0.129	18743	-0.013	0.058	0.817	0.239	18716
rs7498665	0.052	0.054	0.334	0	18745	-0.021	0.042	0.624	0.212	18718
rs1424233	0.031	0.054	0.557	0	18745	-0.040	0.041	0.327	0	18718
rs1805081	0.008	0.053	0.875	0.358	18744	-0.006	0.041	0.875	0.188	18717
rs17782313	-0.072	0.063	0.248	0.467	18737	0.039	0.048	0.413	0	18710
rs29941	0.102	0.057	0.074	0.182	18744	0.035	0.044	0.436	0.133	18717
rs11084753	0.115	0.058	**0.048**	0.404	18739	0.072	0.046	0.114	0.454	18712
rs2287019	-0.049	0.081	0.550	0.124	14954	0.053	0.069	0.448	0.108	14927
rs3810291	-0.104	0.064	0.105	0	18745	-0.096	0.048	**0.045**	0.234	18718
rs6013029	0.107	0.144	0.454	0	15105	-0.009	0.088	0.916	0.016	15082
rs4823006	0.080	0.055	0.145	0	18721	0.029	0.043	0.492	0	18694
