# Supplementary material for: Genome-Wide Association Study to Identify Common Variants Associated with Brachial Circumference: A Meta-Analysis of 14 Cohorts
Source: PLoS One. 2012 Mar 29;7(3):e31369. doi: 10.1371/journal.pone.0031369 (PMC3315559; doi:10.1371/journal.pone.0031369)
Supplement: Table S7 — Association of established T2D loci. CHR - chromosome; POS - position; EA - effect allele; NEA - non-effect allele; EAF - effect allele frequency; SE- standard error; P - p-value; I2- measure of heterogeneity; N - total number of samples (p-values<0.05 in bold).* SNP rs7564886 is a proxy for originally associated T2D SNP rs7578597 (r2 = 1). (PDF) [file pone.0031369.s010.pdf]

Table S7. Association of established T2D loci

SNP information								
**SNP**	**GENE**	**TRAIT**	**REFERENCE**	**CHR**	**POS**	**EA**	**NEA**	**EAF**
rs10923931	*NOTCH2*	T2D	Voight et al. 2010	1	120319482	T	G	0.145
rs340874	*PROX1*	T2D	Dupuis et al. 2010	1	212225879	T	C	0.468
rs780094	*GCKR*	T2D	Dupuis et al. 2010	2	27594741	T	C	0.423
rs11899863	*THADA*	T2D	Voight et al. 2010	2	43472323	T	C	0.149
rs7564886*	*THADA*	T2D	Voight et al. 2010	2	43586532	T	G	0.153
rs243021	*BCL11A*	T2D	Voight et al. 2010	2	60438323	G	A	0.525
rs7593730	*RBMS1*	T2D	Qi et al. 2010	2	160879700	T	C	0.236
rs3923113	*GRB14*	T2D	Kooner et al. 2011	2	165210095	C	A	0.374
rs7578326	*IRS1*	T2D	Voight et al. 2010	2	226728897	G	A	0.385
rs2943641	*IRS1*	T2D	Voight et al. 2010	2	226801989	T	C	0.377
rs13081389	*PPARG*	T2D	Voight et al. 2010	3	12264800	G	A	0.120
rs1801282	*PPARG*	T2D	Voight et al. 2010	3	12368125	G	C	0.174
rs6780569	*UBE2E2*	T2D	Yamauchi et al. 2010	3	23173488	G	A	0.858
rs6795735	*ADAMTS9*	T2D	Voight et al. 2010	3	64680405	T	C	0.445
rs4607103	*ADAMTS9*	T2D	Voight et al. 2010	3	64686944	T	C	0.291
rs11708067	*ADCY5*	T2D	Dupuis et al. 2010	3	124548468	G	A	0.242
rs1470579	*IGF2BP2*	T2D	Voight et al. 2010	3	187011774	C	A	0.323
rs16861329	*ST6GAL1*	T2D	Kooner et al. 2011	3	188149155	T	C	0.176
rs10010131	*WFS1*	T2D	Voight et al. 2010	4	6343816	G	A	0.587
rs1801214	*WFS1*	T2D	Voight et al. 2010	4	6353923	T	C	0.580
rs4457053	*ZBED3*	T2D	Voight et al. 2010	5	76460705	G	A	0.315
rs7754840	*CDKAL1*	T2D	Voight et al. 2010	6	20769229	G	C	0.664
rs10440833	*CDKAL1*	T2D	Voight et al. 2010	6	20796100	T	A	0.696
rs2191349	*DGKB/TMEM195*	T2D	Dupuis et al. 2010	7	15030834	T	G	0.546
rs864745	*JAZF1*	T2D	Voight et al. 2010	7	28147081	T	C	0.507
rs849134	*JAZF1*	T2D	Voight et al. 2010	7	28162747	G	A	0.491
rs4607517	*GCK*	T2D	Dupuis et al. 2010	7	44202193	G	A	0.791
rs972283	*KLF14*	T2D	Voight et al. 2010	7	130117394	G	A	0.524
rs896854	*TP53INP1*	T2D	Voight et al. 2010	8	96029687	T	C	0.508
rs13266634	*SLC30A8*	T2D	Voight et al. 2010	8	118253964	T	C	0.318
rs3802177	*SLC30A8*	T2D	Voight et al. 2010	8	118254206	G	A	0.682
rs17584499	*PTPRD*	T2D	Tsai et al. 2010	9	8869118	T	C	0.227
rs10965250	*CDKN2A/B*	T2D	Voight et al. 2010	9	22123284	G	A	0.796
rs10811661	*CDKN2A/B*	T2D	Voight et al. 2010	9	22124094	T	C	0.789
rs13292136	*CHCHD9*	T2D	Voight et al. 2010	9	81141948	T	C	0.132
rs12779790	*CDC123/CAMK1D*	T2D	Voight et al. 2010	10	12368016	G	A	0.209
rs1802295	*VPS26A*	T2D	Kooner et al. 2011	10	70601480	T	C	0.336
rs1111875	*HHEX/IDE*	T2D	Voight et al. 2010	10	94452862	T	C	0.423
rs5015480	*HHEX/IDE*	T2D	Voight et al. 2010	10	94455539	T	C	0.424
rs7903146	*TCF7L2*	T2D	Voight et al. 2010	10	114748339	T	C	0.315
rs231362	*KCNQ1*	T2D	Voight et al. 2010	11	2648047	G	A	0.496
rs2237892	*KCNQ1*	T2D	Voight et al. 2010	11	2796327	T	C	0.115
rs163184	*KCNQ1*	T2D	Voight et al. 2010	11	2803645	T	G	0.515
rs2283228	*KCNQ1*	T2D	Unoki et al. 2008	11	2806106	C	A	0.117
rs5215	*KCNJ11*	T2D	Voight et al. 2010	11	17365206	T	C	0.626
rs1552224	*CENTD2*	T2D	Voight et al. 2010	11	72110746	C	A	0.185
rs1387153	*MTNR1B*	T2D	Voight et al. 2010	11	92313476	T	C	0.302
rs10830963	*MTNR1B*	T2D	Voight et al. 2010	11	92348358	G	C	0.307
rs1531343	*HMGA2*	T2D	Voight et al. 2010	12	64461161	G	C	0.842
rs4760790	*TSPAN8/LGR5*	T2D	Voight et al. 2010	12	69921061	G	A	0.687
rs7961581	*TSPAN8/LGR5*	T2D	Voight et al. 2010	12	69949369	T	C	0.703
rs7957197	*HNF1A*	T2D	Voight et al. 2010	12	119945069	T	A	0.777
rs1359790	*SPRY2*	T2D	Shu et al. 2010	13	79615157	G	A	0.709
rs7172432	*C2CD4A/B*	T2D	Yamauchi et al. 2010	15	60183681	G	A	0.415

rs7178572	*HMG20A*	T2D	Kooner et al. 2011	15	75534245	G	A	0.681
rs11634397	*ZFAND6*	T2D	Voight et al. 2010	15	78219277	G	A	0.641
rs2028299	*AP3S2*	T2D	Kooner et al. 2011	15	88175261	C	A	0.306
rs8042680	*PRC1*	T2D	Voight et al. 2010	15	89322341	C	A	0.650
rs9939609	*FTO*	T2D	Voight et al. 2010	16	52378028	T	A	0.565
rs11642841	*FTO*	T2D	Voight et al. 2010	16	52402988	C	A	0.570
rs391300	*SRR*	T2D	Tsai et al. 2010	17	2163008	T	C	0.373
rs757210	*HNF1B(TCF2)*	T2D	Voight et al. 2010	17	33170628	T	C	0.404
rs4430796	*HNF1B(TCF2)*	T2D	Voight et al. 2010	17	33172153	G	A	0.492
rs4812829	*HNF4A*	T2D	Kooner et al. 2011	20	42422681	G	A	0.793

CHR - chromosome; POS - position; EA - effect allele; NEA - non-effect allele; EAF - effect allele frequency; SE-standard error; P - p-value; I^2- measure of heterogeneity; N - total number of samples (p-values<0.05 in bold)

* SNP rs7564886 is a proxy for originally associated T2D SNP rs7578597 (r^2=1)

	WOMEN (age adjusted)					WOMEN (age & BMI adjusted)				
SNP	BETA	SE	P	I^2	N	BETA	SE	P	I^2	N
rs10923931	0.002	0.107	0.988	0	9878	0.156	0.095	0.103	0	9865
rs340874	-0.158	0.066	**0.017**	0.170	9893	-0.187	0.061	**0.002**	0	9880
rs780094	-0.015	0.069	0.832	0.129	9871	-0.047	0.062	0.450	0	9858
rs11899863	0.160	0.104	0.125	0	9892	0.012	0.091	0.894	0.075	9879
rs7564886	0.104	0.104	0.317	0	9893	0.061	0.091	0.507	0.268	9880
rs243021	-0.029	0.070	0.678	0.277	9893	0.028	0.060	0.638	0.120	9880
rs7593730	0.040	0.083	0.634	0.108	9869	-0.024	0.075	0.751	0	9856
rs3923113	0.049	0.070	0.486	0.172	9893	0.047	0.062	0.442	0.209	9880
rs7578326	0.095	0.073	0.191	0.114	9893	-0.082	0.065	0.208	0	9880
rs2943641	0.145	0.070	**0.037**	0	9893	-0.042	0.061	0.491	0	9880
rs13081389	0.227	0.135	0.092	0.174	8642	-0.214	0.112	0.056	0	8630
rs1801282	0.208	0.098	**0.033**	0.158	9893	0.202	0.083	**0.015**	0	9880
rs6780569	0.103	0.122	0.400	0	9892	0.089	0.103	0.384	0	9879
rs6795735	0.126	0.068	0.064	0.274	9696	0.034	0.060	0.568	0.085	9688
rs4607103	0.142	0.077	0.066	0	9878	0.031	0.067	0.639	0	9865
rs11708067	0.006	0.080	0.943	0.361	9881	0.044	0.072	0.538	0.199	9868
rs1470579	0.056	0.073	0.439	0.015	9870	-0.003	0.063	0.966	0	9857
rs16861329	0.079	0.100	0.432	0	8876	0.061	0.086	0.478	0	8863
rs10010131	0.036	0.067	0.593	0	9893	0.087	0.059	0.136	0.112	9880
rs1801214	0.035	0.071	0.624	0	9893	0.076	0.061	0.213	0.263	9880
rs4457053	0.070	0.080	0.380	0	9700	-0.002	0.081	0.977	0	9687
rs7754840	-0.088	0.073	0.224	0	9889	-0.133	0.064	**0.038**	0.059	9876
rs10440833	-0.047	0.074	0.524	0	9892	-0.155	0.066	**0.018**	0	9879
rs2191349	-0.088	0.067	0.190	0	9893	-0.023	0.059	0.698	0.332	9880
rs864745	-0.019	0.066	0.769	0	9873	0.114	0.060	0.057	0	9860
rs849134	0.009	0.066	0.891	0	9893	-0.118	0.059	**0.047**	0	9880
rs4607517	-0.071	0.088	0.420	0	9893	-0.112	0.080	0.163	0	9880
rs972283	0.028	0.071	0.696	0	9893	0.034	0.059	0.567	0.178	9880
rs896854	-0.113	0.068	0.097	0.021	9893	-0.100	0.060	0.093	0.123	9880
rs13266634	0.004	0.075	0.957	0.227	9070	-0.102	0.065	0.115	0.316	9057
rs3802177	-0.006	0.075	0.937	0.223	9070	0.102	0.065	0.116	0.314	9057
rs17584499	0.179	0.091	**0.049**	0	9892	0.122	0.082	0.135	0.119	9879
rs10965250	0.188	0.093	**0.043**	0.021	9893	0.068	0.082	0.407	0	9880
rs10811661	0.202	0.092	**0.028**	0	9874	0.069	0.081	0.396	0	9861
rs13292136	-0.075	0.138	0.588	0.306	7365	-0.188	0.121	0.120	0.107	7352
rs12779790	0.087	0.085	0.306	0	9893	0.143	0.079	0.070	0	9880
rs1802295	-0.089	0.073	0.222	0.054	9882	0.027	0.066	0.681	0.2757	9869
rs1111875	0.109	0.068	0.108	0.124	9891	0.066	0.060	0.271	0	9878
rs5015480	0.111	0.068	0.104	0.146	9843	0.070	0.060	0.244	0	9830
rs7903146	-0.008	0.073	0.910	0.136	9892	0.075	0.067	0.263	0.369	9879
rs231362	-0.052	0.069	0.447	0	9893	0.001	0.064	0.992	0	9880
rs2237892	0.090	0.142	0.526	0	8725	-0.041	0.124	0.743	0	8712
rs163184	0.005	0.067	0.938	0.077	9893	0.051	0.061	0.401	0.039	9880
rs2283228	0.103	0.141	0.463	0.027	8725	0.007	0.123	0.957	0	8712
rs5215	0.020	0.069	0.772	0.567	9869	0.016	0.062	0.801	0.091	9856
rs1552224	0.007	0.093	0.940	0	9892	-0.026	0.082	0.750	0.020	9879
rs1387153	-0.028	0.075	0.706	0	9893	0.124	0.067	0.063	0.053	9880
rs10830963	-0.095	0.084	0.257	0	9881	0.103	0.071	0.147	0	9868
rs1531343	0.034	0.116	0.772	0.134	9876	-0.058	0.104	0.580	0	9863
rs4760790	0.001	0.074	0.988	0	9893	0.037	0.065	0.574	0.208	9880
rs7961581	0.036	0.079	0.646	0	9888	0.050	0.069	0.470	0.409	9875
rs7957197	-0.126	0.086	0.141	0	9893	0.040	0.079	0.614	0	9880
rs1359790	0.169	0.073	**0.021**	0	9859	0.030	0.067	0.651	0.093	9846
rs7172432	0.007	0.069	0.924	0.602	9893	-0.137	0.061	**0.024**	0.002	9880

rs7178572	0.038	0.074	0.608	0.474	9893	-0.029	0.066	0.664	0.289	9880
rs11634397	-0.103	0.074	0.164	0.303	9890	-0.034	0.064	0.591	0	9877
rs2028299	-0.053	0.073	0.469	0	9892	0.000	0.066	0.996	0.028	9879
rs8042680	0.012	0.072	0.866	0.146	9887	0.027	0.064	0.668	0.011	9874
rs9939609	-0.052	0.035	0.137	0.552	11600	-0.006	0.021	0.769	0.120	11493
rs11642841	-0.011	0.075	0.886	0.529	9892	0.037	0.063	0.557	0.220	9879
rs391300	0.033	0.077	0.667	0.249	9893	-0.040	0.068	0.556	0.268	9880
rs757210	0.089	0.092	0.332	0	5894	-0.197	0.304	0.516	0	5881
rs4430796	0.031	0.090	0.731	0	5894	-0.317	0.304	0.297	0	5881
rs4812829	-0.052	0.090	0.560	0.08	9892	-0.044	0.078	0.570	0.059	9879

	MEN (age adjusted)					MEN (age & BMI adjusted)				
**SNP**	**BETA**	**SE**	**P**	**I^2**	**N**	**BETA**	**SE**	**P**	**I^2**	**N**
rs10923931	0.043	0.145	0.764	0	8838	0.077	0.093	0.404	0	8824
rs340874	-0.027	0.087	0.758	0.219	8851	-0.006	0.056	0.908	0.224	8837
rs780094	-0.088	0.089	0.320	0.423	8834	-0.065	0.057	0.255	0	8820
rs11899863	0.163	0.136	0.229	0.386	8852	0.280	0.087	**0.001**	0.243	8838
rs7564886	0.151	0.135	0.264	0.175	8852	0.219	0.087	**0.012**	0.129	8838
rs243021	0.103	0.088	0.242	0.475	8852	0.016	0.056	0.777	0.088	8838
rs7593730	0.070	0.114	0.536	0.331	8828	-0.048	0.073	0.512	0	8814
rs3923113	-0.054	0.091	0.556	0	8851	-0.021	0.059	0.719	0	8837
rs7578326	-0.052	0.096	0.583	0.183	8852	-0.052	0.061	0.394	0.1304	8838
rs2943641	0.020	0.091	0.825	0.196	8852	-0.004	0.058	0.946	0	8838
rs13081389	-0.080	0.166	0.628	0	7468	0.035	0.106	0.740	0	7532
rs1801282	0.062	0.121	0.607	0	8852	0.000	0.078	0.998	0.375	8838
rs6780569	-0.017	0.150	0.911	0.415	8852	-0.125	0.096	0.195	0.280	8838
rs6795735	0.172	0.088	**0.050**	0.165	8681	0.044	0.056	0.437	0.081	8675
rs4607103	0.072	0.101	0.472	0.462	8845	-0.012	0.064	0.847	0.405	8831
rs11708067	-0.018	0.106	0.866	0.388	8841	-0.104	0.068	0.123	0	8827
rs1470579	0.103	0.095	0.280	0.149	8817	0.042	0.061	0.489	0	8803
rs16861329	0.194	0.125	0.122	0.209	7864	0.008	0.080	0.924	0.3684	7850
rs10010131	-0.097	0.090	0.283	0.204	8852	-0.050	0.058	0.386	0	8838
rs1801214	-0.150	0.094	0.110	0	8852	-0.077	0.060	0.201	0	8838
rs4457053	-0.257	0.122	**0.035**	0.009	8676	-0.140	0.078	0.071	0.483	8662
rs7754840	0.027	0.093	0.771	0	8847	0.045	0.060	0.452	0.127	8833
rs10440833	0.089	0.098	0.367	0	8852	0.051	0.063	0.418	0.409	8838
rs2191349	-0.005	0.087	0.952	0	8852	-0.019	0.056	0.734	0.262	8838
rs864745	-0.058	0.089	0.517	0.075	8834	0.052	0.057	0.366	0.359	8820
rs849134	0.060	0.089	0.499	0.074	8852	-0.045	0.057	0.430	0.413	8838
rs4607517	-0.145	0.121	0.230	0.301	8852	-0.119	0.077	0.121	0	8838
rs972283	-0.103	0.087	0.235	0	8852	-0.110	0.056	**0.048**	0	8838
rs896854	0.000	0.089	0.999	0.085	8852	0.066	0.057	0.243	0.183	8838
rs13266634	0.129	0.094	0.171	0	8041	-0.099	0.060	0.101	0.405	8027
rs3802177	-0.129	0.094	0.170	0	8040	0.099	0.060	0.101	0.407	8026
rs17584499	0.166	0.121	0.171	0	8851	0.002	0.077	0.984	0	8837
rs10965250	0.157	0.121	0.192	0.149	8851	0.035	0.077	0.650	0.040	8837
rs10811661	0.161	0.118	0.171	0.229	8839	0.039	0.076	0.601	0.118	8825
rs13292136	0.055	0.178	0.759	0	6939	-0.028	0.114	0.804	0	6934
rs12779790	0.039	0.116	0.738	0	8851	0.047	0.074	0.530	0.2364	8837
rs1802295	0.063	0.098	0.524	0	8844	-0.033	0.063	0.600	0	8830
rs1111875	0.000	0.090	0.996	0	8850	-0.048	0.057	0.399	0.054	8836
rs5015480	0.007	0.090	0.940	0	8808	-0.049	0.057	0.392	0.082	8794
rs7903146	-0.230	0.099	**0.021**	0	8852	-0.132	0.063	**0.036**	0.217	8838
rs231362	0.083	0.094	0.378	0.123	8852	0.016	0.060	0.797	0.168	8838
rs2237892	0.174	0.193	0.368	0.336	8257	0.091	0.124	0.462	0.385	8247
rs163184	-0.170	0.090	0.059	0	8852	-0.033	0.057	0.560	0	8838
rs2283228	0.184	0.187	0.326	0.462	8255	0.075	0.120	0.535	0.399	8245
rs5215	0.093	0.091	0.307	0	8833	0.071	0.058	0.222	0.270	8819
rs1552224	0.267	0.123	**0.030**	0	8852	0.067	0.079	0.398	0	8838
rs1387153	0.271	0.098	**0.006**	0	8852	0.128	0.063	**0.042**	0	8838
rs10830963	0.212	0.106	**0.045**	0	8835	0.127	0.068	0.060	0.204	8821
rs1531343	0.052	0.151	0.728	0.553	8822	-0.034	0.097	0.727	0.185	8808
rs4760790	0.009	0.099	0.927	0	8851	-0.017	0.063	0.792	0	8837
rs7961581	0.007	0.104	0.946	0	8846	-0.007	0.067	0.918	0	8832
rs7957197	-0.052	0.113	0.648	0	8852	-0.015	0.072	0.837	0	8838
rs1359790	-0.075	0.101	0.454	0.409	8816	-0.052	0.064	0.414	0.553	8802
rs7172432	-0.007	0.089	0.941	0	8852	0.019	0.057	0.742	0	8838

rs7178572	0.146	0.096	0.130	0	8852	0.080	0.062	0.195	0	8838
rs11634397	0.035	0.095	0.711	0.021	8851	-0.049	0.061	0.419	0	8837
rs2028299	-0.094	0.094	0.318	0	8852	0.022	0.060	0.719	0	8838
rs8042680	-0.065	0.097	0.503	0	8851	-0.012	0.062	0.843	0	8837
rs9939609	-0.113	0.054	**0.036**	0.545	9734	-0.014	0.033	0.656	0.413	9719
rs11642841	-0.186	0.090	**0.039**	0.549	8852	-0.011	0.058	0.844	0.408	8838
rs391300	-0.052	0.102	0.612	0	8852	0.006	0.065	0.924	0.183	8838
rs757210	0.072	0.665	0.913	0	4990	-0.229	0.327	0.485	0	4976
rs4430796	0.505	0.659	0.443	0	4990	0.083	0.327	0.799	0	4976
rs4812829	-0.070	0.118	0.556	0.404	8851	-0.039	0.076	0.606	0.443	8837

	COMBINED (age adjusted)					COMBINED (age & BMI adjusted)				
SNP	BETA	SE	P	I^2	N	BETA	SE	P	I^2	N
rs10923931	0.017	0.086	0.841	0	18716	0.116	0.067	0.082	0	18689
rs340874	-0.110	0.053	**0.036**	0.229	18744	-0.088	0.041	**0.033**	0.038	18717
rs780094	-0.042	0.055	0.438	0.314	18705	-0.056	0.042	0.178	0	18678
rs11899863	0.161	0.083	0.051	0.152	18744	0.152	0.063	**0.015**	0.275	18717
rs7564886	0.121	0.082	0.140	0.013	18745	0.143	0.063	**0.022**	0.237	18718
rs243021	0.022	0.055	0.683	0.396	18745	0.021	0.041	0.603	0.005	18718
rs7593730	0.049	0.067	0.462	0.063	18697	-0.038	0.052	0.471	0	18670
rs3923113	0.011	0.056	0.849	0.092	18744	0.012	0.042	0.783	0	18717
rs7578326	0.041	0.058	0.475	0.188	18745	-0.065	0.044	0.141	0	18718
rs2943641	0.100	0.055	0.071	0.124	18745	-0.022	0.042	0.606	0	18718
rs13081389	0.170	0.105	0.105	0	16110	-0.082	0.077	0.287	0	16162
rs1801282	0.151	0.076	**0.048**	0.026	18745	0.094	0.057	0.098	0.229	18718
rs6780569	0.057	0.095	0.548	0.116	18744	-0.025	0.070	0.722	0	18717
rs6795735	0.144	0.054	**0.007**	0.220	18377	0.039	0.041	0.345	0.078	18363
rs4607103	0.117	0.061	0.057	0.167	18723	0.008	0.046	0.864	0.175	18696
rs11708067	-0.002	0.064	0.978	0.324	18722	-0.033	0.049	0.507	0.112	18695
rs1470579	0.074	0.058	0.202	0.074	18687	0.019	0.044	0.664	0	18660
rs16861329	0.124	0.078	0.114	0	16740	0.032	0.058	0.583	0.132	16713
rs10010131	-0.011	0.054	0.837	0	18745	0.017	0.041	0.673	0	18718
rs1801214	-0.032	0.057	0.570	0	18745	-0.002	0.043	0.967	0.010	18718
rs4457053	-0.028	0.067	0.671	0.049	18376	-0.074	0.056	0.184	0.203	18349
rs7754840	-0.045	0.057	0.433	0	18736	-0.038	0.044	0.381	0.210	18709
rs10440833	0.002	0.059	0.973	0	18744	-0.048	0.045	0.289	0.261	18717
rs2191349	-0.057	0.053	0.283	0	18745	-0.021	0.041	0.604	0.292	18718
rs864745	-0.034	0.053	0.528	0	18707	0.080	0.041	0.052	0	18680
rs849134	0.028	0.053	0.602	0	18745	-0.079	0.041	0.054	0.035	18718
rs4607517	-0.097	0.071	0.173	0.051	18745	-0.115	0.056	**0.039**	0	18718
rs972283	-0.024	0.055	0.658	0	18745	-0.043	0.041	0.285	0.072	18718
rs896854	-0.071	0.054	0.188	0.083	18745	-0.013	0.041	0.746	0.225	18718
rs13266634	0.052	0.059	0.371	0	17111	-0.100	0.044	**0.023**	0.362	17084
rs3802177	-0.054	0.059	0.359	0	17110	0.100	0.044	**0.024**	0.363	17083
rs17584499	0.174	0.073	**0.017**	0	18743	0.058	0.056	0.297	0	18716
rs10965250	0.177	0.074	**0.016**	0.028	18744	0.051	0.056	0.361	0	18717
rs10811661	0.187	0.073	**0.010**	0.006	18713	0.054	0.055	0.330	0	18686
rs13292136	-0.026	0.109	0.814	0	14304	-0.102	0.083	0.218	0	14286
rs12779790	0.071	0.069	0.297	0	18744	0.090	0.054	0.095	0.128	18717
rs1802295	-0.035	0.059	0.548	0	18726	-0.004	0.045	0.927	0	18699
rs1111875	0.070	0.054	0.198	0	18741	0.006	0.041	0.890	0	18714
rs5015480	0.073	0.054	0.178	0	18651	0.007	0.041	0.863	0	18624
rs7903146	-0.087	0.059	0.139	0.138	18744	-0.035	0.046	0.450	0.3878	18717
rs231362	-0.004	0.055	0.939	0	18745	0.008	0.044	0.852	0	18718
rs2237892	-0.004	0.114	0.969	0.213	16982	0.025	0.088	0.773	0.061	16959
rs163184	-0.058	0.054	0.285	0.097	18745	0.007	0.042	0.876	0	18718
rs2283228	-0.002	0.113	0.983	0.338	16980	0.041	0.086	0.630	0.153	16957
rs5215	0.047	0.055	0.391	0.378	18702	0.044	0.042	0.297	0.206	18675
rs1552224	0.102	0.074	0.171	0	18744	0.023	0.057	0.680	0.003	18717
rs1387153	0.081	0.059	0.170	0	18745	0.127	0.046	**0.006**	0	18718
rs10830963	0.023	0.066	0.723	0	18716	0.117	0.049	**0.018**	0	18689
rs1531343	0.041	0.092	0.659	0.41	18698	-0.045	0.071	0.528	0.039	18671
rs4760790	0.004	0.059	0.951	0	18744	0.008	0.045	0.856	0.047	18717
rs7961581	0.025	0.063	0.690	0	18734	0.019	0.048	0.687	0.180	18707
rs7957197	-0.098	0.068	0.151	0	18745	0.011	0.053	0.837	0	18718
rs1359790	0.085	0.059	0.150	0.312	18675	-0.013	0.046	0.782	0.41	18648
rs7172432	0.002	0.054	0.973	0.377	18745	-0.054	0.042	0.193	0	18718

rs7178572	0.078	0.059	0.186	0.235	18745	0.029	0.045	0.517	0.021	18718
rs11634397	-0.050	0.059	0.389	0.217	18741	-0.043	0.044	0.335	0	18714
rs2028299	-0.068	0.058	0.241	0	18744	0.013	0.044	0.775	0	18717
rs8042680	-0.015	0.058	0.791	0.071	18738	0.007	0.044	0.878	0	18711
rs9939609	-0.073	0.029	**0.013**	0.605	21334	-0.011	0.019	0.571	0.321	21306
rs11642841	-0.082	0.058	0.154	0.560	18744	0.011	0.043	0.800	0.329	18717
rs391300	0.002	0.062	0.973	0	18745	-0.016	0.047	0.732	0.232	18718
rs757210	0.089	0.091	0.330	0	10884	-0.201	0.224	0.367	0	10857
rs4430796	0.040	0.089	0.655	0	10884	-0.132	0.223	0.555	0	10857
rs4812829	-0.059	0.071	0.409	0.276	18743	-0.042	0.054	0.440	0.295	18716
