# Supplementary material for: Genome-Wide Association Study to Identify Common Variants Associated with Brachial Circumference: A Meta-Analysis of 14 Cohorts
Source: PLoS One. 2012 Mar 29;7(3):e31369. doi: 10.1371/journal.pone.0031369 (PMC3315559; doi:10.1371/journal.pone.0031369)
Supplement: Table S8 — Association of loci influencing glycemic traits. CHR - chromosome; POS - position; EA - effect allele; NEA - non-effect allele; EAF - effect allele frequency; SE- standard error; P - p-value; I2- measure of heterogeneity; N - total number of samples (p-values<0.05 in bold);FGlu - fasting glucose; FIns - fasting insulin; 2 hrGlu - 2 h after glucose challenge; HbA1C - glycated hemoglobin. (PDF) [file pone.0031369.s011.pdf]

Table S8. Association of loci influencing glycemic traits

SNP information								
**SNP**	**GENE**	**TRAIT**	**REFERENCE**	**CHR**	**POS**	**EA**	**NEA**	**EAF**
rs9727115	*SNX7*	Fasting Proinsulin	Strawbridge et al. 2011	1	98949841	G	A	0.630
rs340874	*PROX1*	FGlu	Dupiuis et al. 2010	1	212225879	T	C	0.468
rs1260326	*GCKR*	2hrGlu	Saxena et al. 2010	2	27584444	T	C	0.432
rs780094	*GCKR*	Fglu/Fins	Dupiuis et al. 2010	2	27594741	T	C	0.423
rs560887	*G6PC2*	FGlu	Dupiuis et al. 2010	2	169471394	T	C	0.322
rs552976	*ABCB11/G6PC2*	HbA1C	Soranzo et al. 2010	2	169499684	G	A	0.621
rs11708067	*ADCY5*	FGlu	Dupiuis et al. 2010	3	124548468	G	A	0.242
rs2877716	*ADCY5*	2hrGlu	Saxena et al. 2010	3	124577141	T	C	0.262
rs11920090	*SLC2A2*	FGlu	Dupiuis et al. 2010	3	172200215	T	A	0.824
rs6235	*PCSK1*	Fasting Proinsulin	Strawbridge et al. 2011	5	95754654	G	C	0.307
rs2191349	*DGKB/TMEM195*	FGlu	Dupiuis et al. 2010	7	15030834	T	G	0.546
rs1799884	*GCK*	HbA1C	Soranzo et al. 2010	7	44195593	T	C	0.209
rs4607517	*GCK*	FGlu	Dupiuis et al. 2010	7	44202193	G	A	0.791
rs4737009	*ANK1*	HbA1C	Soranzo et al. 2010	8	41749562	G	A	0.722
rs11558471	*SLC30A8*	Fglu/Fasting proinsulin	Dupiuis et al. 2010	8	118254914	G	A	0.321
rs7034200	*GLIS3*	FGlu	Dupiuis et al. 2010	9	4279050	C	A	0.497
rs16926246	*HK1;HK1-tc*	HbA1C	Soranzo et al. 2010	10	70763398	T	C	0.147
rs10885122	*ADRA2A*	FGlu	Dupiuis et al. 2010	10	113032083	T	G	0.154
rs4506565	*TCF7L2*	FGlu	Dupiuis et al. 2010	10	114746031	T	A	0.332
rs7903146	*TCF7L2*	Fasting Proinsulin	Strawbridge et al. 2011	10	114748339	T	C	0.315
rs12243326	*TCF7L2*	2hrGlu	Saxena et al. 2010	10	114778805	T	C	0.698
rs11605924	*CRY2*	FGlu	Dupiuis et al. 2010	11	45829667	C	A	0.512
rs10501320	*MADD*	Fasting Proinsulin	Strawbridge et al. 2011	11	47250375	G	C	0.681
rs10838687	*MADD *2nd signal*	Fasting Proinsulin	Strawbridge et al. 2011	11	47269468	T	G	0.761
rs7944584	*MADD*	FGlu	Dupiuis et al. 2010	11	47292896	T	A	0.324
rs174550	*FADS1*	FGlu	Dupiuis et al. 2010	11	61328054	T	C	0.657
rs11603334	*ARAP1*	Fasting Proinsulin	Strawbridge et al. 2011	11	72110633	G	A	0.815
rs1387153	*MTNR1B*	HbA1C	Soranzo et al. 2010	11	92313476	T	C	0.302
rs10830963	*MTNR1B*	FGlu	Dupiuis et al. 2010	11	92348358	G	C	0.307
rs35767	*IGF1*	FIns	Dupiuis et al. 2010	12	101399699	G	A	0.806
rs7998202	*ATP11A/ TUBGCP3*	HbA1C	Soranzo et al. 2010	13	112379869	G	A	0.185
rs17271305	*VPS13C*	2hrGlu	Saxena et al. 2010	15	60120272	G	A	0.396
rs4502156	*VPS13C/C2CD4A/B*	Fasting Proinsulin	Strawbridge et al. 2011	15	60170447	T	C	0.582
rs11071657	*FAM148B (C2CD4B)*	FGlu	Dupiuis et al. 2010	15	60221254	G	A	0.369
rs1549318	*LARP6*	Fasting Proinsulin	Strawbridge et al. 2011	15	68896201	T	C	0.599
rs4790333	*SGSM2*	Fasting Proinsulin	Strawbridge et al. 2011	17	2209453	T	C	0.455
rs1046896	*FN3K*	HbA1C	Soranzo et al. 2010	17	78278822	T	C	0.346
rs10423928	*GIPR*	2hrGlu	Saxena et al. 2010	19	50874144	T	A	0.746
rs855791	*TMPRSS6*	HbA1C	Soranzo et al. 2010	22	35792882	G	A	0.555
rs2779116	*SPTA1*	HbA1C	Soranzo et al. 2010	1	156852039	T	C	0.280
rs1800562	*HFE*	HbA1C	Soranzo et al. 2010	6	26201120	G	A	0.800

CHR - chromosome; POS - position; EA - effect allele; NEA - non-effect allele; EAF - effect allele frequency; SE- standard error; P - p-value; I^2- measure of heterogeneity; N - total number of samples (p-values<0.05 in bold)
FGlu - fasting glucose; FIns - fasting insulin; 2hrGlu - 2h after glucose challenge; HbA1C - glycated hemoglobin

	WOMEN (age adjusted)					WOMEN (age & BMI adjusted)				
SNP	BETA	SE	P	I^2	N	BETA	SE	P	I^2	N
rs9727115	0.025	0.070	0.721	0	9893	0.080	0.062	0.199	0.102	9880
rs340874	-0.158	0.066	**0.017**	0.170	9893	-0.187	0.061	**0.002**	0	9880
rs1260326	-0.025	0.068	0.719	0.175	9893	-0.061	0.061	0.316	0	9880
rs780094	-0.015	0.069	0.832	0.129	9871	-0.047	0.062	0.450	0	9858
rs560887	-0.053	0.075	0.481	0.354	9824	-0.097	0.068	0.156	0	9811
rs552976	0.069	0.071	0.335	0.242	9882	0.040	0.062	0.518	0	9869
rs11708067	0.006	0.080	0.943	0.361	9881	0.044	0.072	0.538	0.199	9868
rs2877716	-0.008	0.077	0.913	0	9892	0.058	0.070	0.404	0	9879
rs11920090	-0.038	0.100	0.700	0	9893	-0.079	0.085	0.353	0	9880
rs6235	0.029	0.076	0.702	0.214	9892	-0.030	0.068	0.656	0.300	9879
rs2191349	-0.088	0.067	0.190	0	9893	-0.023	0.059	0.698	0.332	9880
rs1799884	0.064	0.088	0.469	0	9890	0.116	0.080	0.148	0	9877
rs4607517	-0.071	0.088	0.420	0	9893	-0.112	0.080	0.163	0	9880
rs4737009	-0.025	0.082	0.761	0	9885	-0.078	0.071	0.274	0	9872
rs11558471	0.009	0.075	0.906	0.218	9070	-0.103	0.065	0.112	0.359	9057
rs7034200	-0.065	0.066	0.329	0	9893	-0.018	0.060	0.770	0	9880
rs16926246	-0.030	0.111	0.787	0.558	9070	0.044	0.114	0.698	0.205	9057
rs10885122	0.111	0.113	0.325	0.212	9892	0.092	0.089	0.304	0.366	9879
rs4506565	0.015	0.072	0.834	0	9892	0.080	0.065	0.223	0.361	9879
rs7903146	-0.008	0.073	0.910	0.136	9892	0.075	0.067	0.263	0.369	9879
rs12243326	0.021	0.074	0.776	0.204	9885	-0.128	0.068	0.059	0.151	9872
rs11605924	0.039	0.067	0.555	0.361	9893	-0.073	0.059	0.211	0.427	9880
rs10501320	-0.011	0.075	0.888	0.456	9892	-0.064	0.066	0.335	0.670	9879
rs10838687	0.010	0.081	0.897	0.325	9892	0.051	0.069	0.462	0.261	9879
rs7944584	0.034	0.076	0.655	0.463	9876	0.083	0.068	0.220	0.651	9863
rs174550	-0.151	0.071	**0.034**	0.246	9892	0.080	0.062	0.203	0.345	9879
rs11603334	-0.007	0.093	0.941	0	9892	0.026	0.082	0.746	0.044	9879
rs1387153	-0.028	0.075	0.706	0	9893	0.124	0.067	0.063	0.053	9880
rs10830963	-0.095	0.084	0.257	0	9881	0.103	0.071	0.147	0	9868
rs35767	0.073	0.092	0.426	0.063	9892	0.063	0.083	0.446	0.495	9879
rs7998202	0.072	0.102	0.477	0	9892	0.059	0.096	0.539	0	9879
rs17271305	0.040	0.069	0.561	0.302	9893	-0.047	0.061	0.442	0.261	9880
rs4502156	0.005	0.070	0.938	0.610	9893	0.135	0.061	**0.028**	0	9880
rs11071657	-0.010	0.070	0.888	0.270	9893	-0.077	0.062	0.215	0	9880
rs1549318	0.087	0.069	0.207	0	9893	0.054	0.062	0.384	0	9880
rs4790333	0.029	0.068	0.664	0.337	9871	0.102	0.060	0.089	0.461	9858
rs1046896	-0.102	0.076	0.176	0.137	9870	0.000	0.066	0.997	0.275	9857
rs10423928	-0.041	0.091	0.656	0	8877	-0.173	0.092	0.062	0.196	8864
rs855791	0.055	0.068	0.417	0	9892	-0.080	0.061	0.187	0	9879
rs2779116	0.135	0.076	0.076	0.288	9245	0.015	0.070	0.830	0	9232
rs1800562	-0.225	0.188	0.231	0.032	3499	-0.398	0.654	0.543	0	3491

	MEN (age adjusted)					MEN (age & BMI adjusted)				
SNP	BETA	SE	P	I^2	N	BETA	SE	P	I^2	N
rs9727115	0.107	0.092	0.246	0.258	8852	0.088	0.059	0.135	0.048	8838
rs340874	-0.027	0.087	0.758	0.219	8851	-0.006	0.056	0.908	0.224	8837
rs1260326	-0.066	0.089	0.453	0.423	8852	-0.067	0.057	0.236	0	8838
rs780094	-0.088	0.089	0.320	0.423	8834	-0.065	0.057	0.255	0	8820
rs560887	0.092	0.103	0.372	0.104	8792	0.036	0.065	0.586	0	8778
rs552976	-0.011	0.095	0.910	0.334	8847	-0.011	0.061	0.860	0	8833
rs11708067	-0.018	0.106	0.866	0.388	8841	-0.104	0.068	0.123	0	8827
rs2877716	-0.091	0.104	0.383	0.325	8849	-0.148	0.067	**0.026**	0	8835
rs11920090	-0.017	0.126	0.896	0.157	8852	-0.041	0.081	0.614	0.255	8838
rs6235	0.138	0.101	0.174	0.136	8852	0.070	0.065	0.282	0	8838
rs2191349	-0.005	0.087	0.952	0	8852	-0.019	0.056	0.734	0.262	8838
rs1799884	0.148	0.121	0.218	0.204	8851	0.118	0.077	0.125	0	8837
rs4607517	-0.145	0.121	0.230	0.301	8852	-0.119	0.077	0.121	0	8838
rs4737009	0.143	0.103	0.164	0	8843	0.054	0.066	0.413	0.052	8829
rs11558471	0.128	0.094	0.174	0	8041	-0.096	0.060	0.110	0.398	8027
rs7034200	-0.020	0.089	0.819	0.219	8852	0.002	0.057	0.975	0	8838
rs16926246	-0.213	0.166	0.201	0	8040	-0.079	0.105	0.451	0.021	8026
rs10885122	0.041	0.132	0.755	0	8851	0.038	0.084	0.653	0.219	8837
rs4506565	-0.161	0.096	0.093	0	8852	-0.114	0.061	0.063	0.306	8838
rs7903146	-0.230	0.099	**0.021**	0	8852	-0.132	0.063	**0.036**	0.217	8838
rs12243326	0.195	0.101	0.053	0	8836	0.058	0.064	0.369	0.377	8822
rs11605924	0.006	0.087	0.945	0	8851	0.002	0.056	0.976	0	8837
rs10501320	-0.003	0.095	0.976	0.176	8851	0.008	0.061	0.894	0.390	8837
rs10838687	0.033	0.106	0.755	0	8851	0.029	0.068	0.668	0.302	8837
rs7944584	0.032	0.097	0.741	0.182	8833	0.003	0.062	0.967	0.407	8819
rs174550	-0.023	0.095	0.808	0	8851	-0.061	0.060	0.311	0	8837
rs11603334	-0.267	0.123	**0.030**	0	8852	-0.067	0.079	0.396	0	8838
rs1387153	0.271	0.098	**0.006**	0	8852	0.128	0.063	**0.042**	0	8838
rs10830963	0.212	0.106	**0.045**	0	8835	0.127	0.068	0.060	0.204	8821
rs35767	-0.127	0.120	0.290	0	8851	-0.014	0.077	0.858	0.132	8837
rs7998202	0.107	0.139	0.441	0	8676	0.093	0.089	0.296	0	8662
rs17271305	-0.105	0.091	0.251	0	8852	-0.034	0.058	0.564	0	8838
rs4502156	-0.012	0.090	0.896	0	8851	-0.023	0.057	0.689	0	8837
rs11071657	-0.128	0.092	0.167	0.033	8852	-0.086	0.059	0.146	0	8838
rs1549318	0.058	0.091	0.526	0.17	8852	-0.027	0.058	0.641	0	8838
rs4790333	0.118	0.089	0.184	0	8839	0.054	0.057	0.343	0.222	8825
rs1046896	0.104	0.097	0.286	0.214	8832	0.060	0.062	0.330	0	8818
rs10423928	0.202	0.138	0.142	0.552	7864	0.042	0.087	0.632	0.234	7850
rs855791	0.056	0.090	0.532	0	8852	0.013	0.057	0.816	0	8838
rs2779116	0.086	0.100	0.390	0	8330	0.057	0.064	0.377	0	8316
rs1800562	0.181	1.575	0.909	0	3166	-0.015	0.689	0.983	0	3157

	COMBINED (age adjusted)					COMBINED (age & BMI adjusted)				
SNP	BETA	SE	P	I^2	N	BETA	SE	P	I^2	N
rs9727115	0.055	0.056	0.325	0.034	18745	0.084	0.043	**0.050**	0	18718
rs340874	-0.110	0.053	**0.036**	0.229	18744	-0.088	0.041	**0.033**	0.038	18717
rs1260326	-0.040	0.054	0.456	0.316	18745	-0.065	0.042	0.119	0	18718
rs780094	-0.042	0.055	0.438	0.314	18705	-0.056	0.042	0.178	0	18678
rs560887	-0.002	0.061	0.978	0.263	18616	-0.028	0.047	0.553	0	18589
rs552976	0.040	0.057	0.484	0.296	18729	0.014	0.043	0.748	0	18702
rs11708067	-0.002	0.064	0.978	0.324	18722	-0.033	0.049	0.507	0.112	18695
rs2877716	-0.037	0.062	0.554	0.107	18741	-0.048	0.048	0.317	0	18714
rs11920090	-0.030	0.078	0.700	0	18745	-0.058	0.059	0.323	0	18718
rs6235	0.068	0.061	0.264	0.197	18744	0.023	0.047	0.630	0.108	18717
rs2191349	-0.057	0.053	0.283	0	18745	-0.021	0.041	0.604	0.292	18718
rs1799884	0.094	0.071	0.189	0	18741	0.116	0.056	**0.037**	0	18714
rs4607517	-0.097	0.071	0.173	0.051	18745	-0.115	0.056	**0.039**	0	18718
rs4737009	0.041	0.064	0.527	0	18728	-0.006	0.048	0.901	0	18701
rs11558471	0.055	0.059	0.347	0	17111	-0.099	0.044	**0.025**	0.379	17084
rs7034200	-0.048	0.053	0.362	0.090	18745	-0.008	0.041	0.845	0	18718
rs16926246	-0.086	0.093	0.354	0.355	17110	-0.022	0.077	0.772	0.098	17083
rs10885122	0.081	0.086	0.343	0	18743	0.064	0.061	0.300	0.303	18716
rs4506565	-0.049	0.058	0.396	0	18744	-0.023	0.045	0.600	0.407	18717
rs7903146	-0.087	0.059	0.139	0.138	18744	-0.035	0.046	0.450	0.387	18717
rs12243326	0.082	0.059	0.168	0.163	18721	-0.030	0.047	0.524	0.351	18694
rs11605924	0.027	0.053	0.608	0	18744	-0.035	0.040	0.385	0.234	18717
rs10501320	-0.007	0.059	0.905	0.292	18743	-0.025	0.045	0.584	0.545	18716
rs10838687	0.018	0.064	0.781	0	18743	0.040	0.048	0.412	0.073	18716
rs7944584	0.033	0.060	0.585	0.302	18709	0.039	0.046	0.394	0.533	18682
rs174550	-0.104	0.057	0.067	0	18743	0.007	0.043	0.880	0.117	18716
rs11603334	-0.102	0.074	0.171	0	18744	-0.023	0.057	0.681	0.016	18717
rs1387153	0.081	0.059	0.170	0	18745	0.127	0.046	**0.006**	0	18718
rs10830963	0.023	0.066	0.723	0	18716	0.117	0.049	**0.018**	0	18689
rs35767	-0.001	0.073	0.985	0	18743	0.021	0.056	0.705	0.246	18716
rs7998202	0.086	0.082	0.296	0	18568	0.076	0.065	0.243	0	18541
rs17271305	-0.012	0.055	0.828	0.089	18745	-0.041	0.042	0.328	0	18718
rs4502156	-0.001	0.055	0.981	0.407	18744	0.051	0.042	0.222	0	18717
rs11071657	-0.053	0.056	0.346	0.196	18745	-0.082	0.043	0.056	0	18718
rs1549318	0.076	0.055	0.165	0	18745	0.010	0.042	0.804	0	18718
rs4790333	0.062	0.054	0.250	0	18710	0.077	0.041	0.062	0.361	18683
rs1046896	-0.024	0.060	0.690	0.239	18702	0.031	0.045	0.494	0.105	18675
rs10423928	0.033	0.076	0.667	0.369	16741	-0.058	0.063	0.357	0.254	16714
rs855791	0.056	0.054	0.305	0	18744	-0.032	0.042	0.447	0	18717
rs2779116	0.055	0.061	0.359	0.059	17575	0.038	0.047	0.416	0	17548
rs1800562	-0.219	0.186	0.240	0	6665	-0.172	0.476	0.718	0	6648
